# Quantification of High-dimensional Non-Gaussianities and Its Implication to Fisher Analysis in Cosmology

**DOI:** 10.3847/1538-4357/acbe3b

**Published:** 2023-04-05

**Authors:** Core Francisco Park, Erwan Allys, Francisco Villaescusa-Navarro, Douglas Finkbeiner

**Affiliations:** 1 Harvard University, 17 Oxford Street, Cambridge, MA 02138, USA; corefranciscopark@g.harvard.edu; 2 Laboratoire de Physique de l’École Normale Supérieure, ENS, Université PSL, CNRS, Sorbonne Université, Université Paris Cité, F-75005 Paris, France; 3 Center for Computational Astrophysics, Flatiron Institute, 162 5th Avenue, New York, NY 10010, USA; 4 Department of Astrophysical Sciences, Princeton University, Peyton Hall, Princeton, NJ 08544, USA

## Abstract

It is well known that the power spectrum is not able to fully characterize the statistical properties of non-Gaussian density fields. Recently, many different statistics have been proposed to extract information from non-Gaussian cosmological fields that perform better than the power spectrum. The Fisher matrix formalism is commonly used to quantify the accuracy with which a given statistic can constrain the value of the cosmological parameters. However, these calculations typically rely on the assumption that the sampling distribution of the considered statistic follows a multivariate Gaussian distribution. In this work, we follow Sellentin & Heavens and use two different statistical tests to identify non-Gaussianities in different statistics such as the power spectrum, bispectrum, marked power spectrum, and wavelet scattering transform (WST). We remove the non-Gaussian components of the different statistics and perform Fisher matrix calculations with the *Gaussianized* statistics using Quijote simulations. We show that constraints on the parameters can change by a factor of ∼2 in some cases. We show with simple examples how statistics that do not follow a multivariate Gaussian distribution can achieve artificially tight bounds on the cosmological parameters when using the Fisher matrix formalism. We think that the non-Gaussian tests used in this work represent a powerful tool to quantify the robustness of Fisher matrix calculations and their underlying assumptions. We release the code used to compute the power spectra, bispectra, and WST that can be run on both CPUs and GPUs.

## 1. Introduction

Upcoming surveys of the large-scale structure (LSS) of the universe like DESI (Levi et al. 2013), Euclid (Laureijs et al. 2011), and the Rubin Observatory (LSST Science Collaboration et al. 2009; LSST Dark Energy Science Collaboration 2012; Jain et al. 2015) will map the distribution of galaxies in angular and redshift space over large cosmological volumes. These galaxies will serve as a biased tracer of the underlying matter density field. If this field were an homogeneous Gaussian random field, the power spectrum would contain all of the information about the cosmological parameters. However, the matter density field today or at low redshift is highly non-Gaussian, especially at the small scales (≲10 *h* ^−1^ Mpc), and the power spectrum is not able to fully characterize the statistical properties of it.

Recently, different methods and statistics have been developed to efficiently extract the cosmological information hidden in the matter, halo, and galaxy density fields (Neyrinck et al. 2009; Simpson et al. 2011, 2013; Coulton et al. 2019; Liu & Madhavacheril 2019; Li et al. 2019; Marques et al. 2019; Vicinanza et al. 2019; Ajani et al. 2020; Allys et al. 2020; Banerjee et al. 2020; Dai et al. 2020; de la Bella et al. 2021; Friedrich et al. 2020; Giri & Smith 2022; Gualdi et al. 2021a, 2021b; Hahn et al. 2020; Lee & Ryu 2020; Ryu & Lee 2020; Villaescusa-Navarro et al. 2020; Valogiannis & Dvorkin 2022; Uhlemann et al. 2020; Zhang et al. 2020; Banerjee & Abel 2021a, 2021b; Bayer et al. 2021; Cheng & Menard 2021a; Hahn & Villaescusa-Navarro 2021; Harnois-Deraps et al. 2021, 2022; Kuruvilla 2022; Kuruvilla & Aghanim 2021; Massara et al. 2021; Naidoo et al. 2022; Porth et al. 2023; Samushia et al. 2021; Liu et al. 2022). For instance, Hahn et al. (2020) uses the halo bispectrum to break the parameter degeneracy between *σ*_8_ and *M*_*ν*_ and shows that the sum of neutrino masses can be measured with ∼5× higher precision than just using the power spectrum. Vicinanza et al. (2019) evaluates the Minkowski functionals of lensing convergence maps, which are helpful breaking the Ω_*m*_−*σ*_8_ degeneracy. Other promising approaches consist of applying a simple nonlinear input transform to the density field. Simpson et al. (2011, 2013) clips the density field to a maximum value to reduce the large contribution of massive halos to the power spectrum, while Neyrinck et al. (2009) log transforms the density field to weight all elements of the cosmic web in a similar manner. Massara et al. (2021) showed that the marked power spectrum, conceptually similar to a density field transformation, sets greatly improved constraints on all cosmological parameters.

In recent years, new methods applying nonlinear operators on top of wavelet transforms, the so-called wavelet scattering transform (WST; Bruna & Mallat 2013), have also obtained promising results (Cheng et al. 2020; Cheng & Ménard 2021a; Valogiannis & Dvorkin 2022). Valogiannis & Dvorkin (2022) for instance suggested that the WST can improve constraints on the value of the cosmological parameters by a factor between 3 and 100 better than the power spectrum, when evaluated on the three-dimensional matter density field. A similar method called the Wavelet Phase Harmonics has been introduced in Allys et al. (2020), showing very promising results in terms of information content.^5^ 
^5^
In this work, we use both information content and parameter constraints. Higher information content means tighter parameter constraints, and vice versa.


It is a standard practice in cosmology to quantify the information content a given statistic carries by using the Fisher matrix formalism. For instance, the Quijote simulations (Villaescusa-Navarro et al. 2020), a suite of 44100 full *N*-body simulations, was designed to perform Fisher matrix calculations, and several of the works listed above employ such simulations to address this point.

Although conceptually simple, the standard Fisher matrix analyses rely on some assumptions like the Gaussianity of the considered statistic. In this work, we investigate the level of non-Gaussianities of different statistics and their impact on Fisher matrix calculations. Overall, we argue how the use of several statistical tools can help in the quest to find optimal and robust statistics to extract the maximum information from the cosmic web and its tracers.

The rest of the paper is organized as follows:

1. First, in Section 2 we introduce the Fisher matrix formalism and two statistical tests to quantify the level of non-Gaussianity in a given statistic. We also propose a method to remove non-Gaussian components from the considered statistic.
2. Second, in Section 3 we illustrate the problem by considering the power spectrum and some statistics derived from it and show how the Fisher matrix formalism can give different results just a result of transformations that do not carry cosmological information. We show how to ameliorate these situations by making use of the non-Gaussian tests.
3. Third, we repeat the above exercise but for other statistics of the LSS of the universe such as the bispectrum, marked power spectrum, and WST in Section 4.
4. Next, we describe the limitations of the tools used to identify non-Gaussianities in Section 5.
5. Finally, we draw our conclusions in Section 6.

## 2. The Fisher Matrix Formalism and Gaussianity Tests

In this section we first describe the Fisher matrix formalism and then we discuss two different tests to identify non-Gaussianities in a given statistics. We then describe a method to remove non-Gaussian dimensions from generic statistics. We note that while in this paper we focus our attention on cosmology, these methods are generic and can therefore be applied to problems outside cosmology.

### 2.1. Fisher Matrix Formalism

The Fisher matrix formalism (Fisher 1922; Cover & Thomas 2006) is a method to quantify the accuracy that a given statistic can constrain the value of some parameters. The Fisher matrix formalism is commonly used in cosmology to quantify the accuracy that a given statistic can place on the value of the cosmological parameters. One of its big advantages is that is does not require actual data to perform the calculation.

When having *N* parameters, ${\boldsymbol{\theta }}\in {{ \mathcal R }}^{N}$, conditioning the value of a statistic ***X***, the Fisher information can be represented in a matrix form as:

\begin{eqnarray*}{F}_{{ij}}({\boldsymbol{\theta }})={E}_{{\boldsymbol{X}}}\left[\left(\displaystyle \frac{\partial }{\partial {\theta }_{i}}\mathrm{log}\,{ \mathcal L }({\boldsymbol{X}};{\boldsymbol{\theta }})\right)\left(\displaystyle \frac{\partial }{\partial {\theta }_{j}}\mathrm{log}\,{ \mathcal L }({\boldsymbol{X}};{\boldsymbol{\theta }})\right)| {\boldsymbol{\theta }}\right],\end{eqnarray*}

where ${ \mathcal L }({\boldsymbol{X}};{\boldsymbol{\theta }})$ is the likelihood function. Formally, the likelihood function is a function of ***θ*** when the observed sample ***X*** is fixed. However, in this work, we will explore the case where ***θ*** is fixed instead. We will call ${ \mathcal L }({\boldsymbol{X}};{\boldsymbol{\theta }})$, the probability of ***X*** when ***θ*** is fixed, the sampling distribution. When the likelihood can be differentiated twice, this can be rewritten as

\begin{eqnarray*}{F}_{{ij}}({\boldsymbol{\theta }})=-{E}_{{\boldsymbol{X}}}\left[\displaystyle \frac{{\partial }^{2}}{\partial {\theta }_{i}\partial {\theta }_{j}}\mathrm{log}\,{ \mathcal L }({\boldsymbol{X}};{\boldsymbol{\theta }})| {\boldsymbol{\theta }}\right].\end{eqnarray*}

This matrix is called the Fisher information matrix (FIM; Fisher 1922; Cover & Thomas 2006). The Cramer–Rao theorem states that the variance of an optimal unbiased estimator on the parameter *θ*_*i*_ will satisfy

\begin{eqnarray*}{\delta }^{2}{\theta }_{i}\geqslant {({F}^{-1})}_{{ii}}.\end{eqnarray*}

When the likelihood ${ \mathcal L }({\boldsymbol{X}};{\boldsymbol{\theta }})$ is a multivariate Gaussian distribution, the FIM can be expressed as (see, e.g., Tegmark et al. 1997)

\begin{eqnarray*}{F}_{{ij}}^{\theta }=\frac{\partial {\mu }_{k}}{\partial {\theta }_{i}}\frac{\partial {\mu }_{l}}{\partial {\theta }_{j}}{{\mathrm{\Sigma }}}_{{kl}}^{-1}+\frac{1}{2}{{\mathrm{\Sigma }}}_{{kl}}^{-1}\frac{\partial {{\mathrm{\Sigma }}}_{{lm}}}{\partial {\theta }_{i}}{{\mathrm{\Sigma }}}_{{mn}}^{-1}\frac{\partial {{\mathrm{\Sigma }}}_{nk}}{\partial {\theta }_{j}},\end{eqnarray*}

where *μ* and Σ are the mean and the covariance of the considered statistic. In this equation and in the whole paper, we assume Einstein notation. Following Carron (2013), we only keep the first term in this equation since we are using a non-Gaussian distributed estimator, and this term will lead to overestimating the Fisher information. This has been shown explicitly by Carron (2013) for Gaussian fields, but we conservatively apply this in our case where we have non-Gaussian fields. We do not come back on this hypothesis in the present paper. The Fisher matrix is then further simplified as:

\begin{eqnarray*}{F}_{{ij}}^{\theta }=\displaystyle \frac{\partial {\mu }_{k}}{\partial {\theta }_{i}}\displaystyle \frac{\partial {\mu }_{l}}{\partial {\theta }_{j}}{{\mathrm{\Sigma }}}_{{kl}}^{-1}.\end{eqnarray*}

To evaluate the FIM (e.g., from numerical simulations), two ingredients are needed:

1. Estimate the covariance Σ of the statistic, which can be computed from many independent realizations, at a fixed value of the cosmological parameters, of the considered statistic.
2. Estimate the partial derivatives of the expectation value of the statistic with respect to the parameters.

In theory, this is enough to evaluate the FIM and to derive optimal constraints on the cosmological parameters from Equation (3). In practice, however, there are a few subtleties to this analysis, such as:

1. The estimated covariance and/or derivatives might have not numerically converged.
2. Numerical precision can affect calculation of derivatives and matrix inversion.
3. Spurious effects may arise due to artifacts from the way the statistic is represented.
4. Noise and systematics may not have to be taken into account.
5. The sampling distribution of the considered statistic can be substantially non-Gaussian.

It is common practice to perform some sanity checks to verify that the first and second points above are not a problem. There are also standard practices to investigate the effects of the third. While including noise may be easy, systematics may be more challenging. In this work however, we focus our attention on the last point, that it is usually not taken into account, and it is commonly assumed that the sampling distribution is a multivariate Gaussian distribution.

#### 2.1.1. Standard Fisher Analysis

We will start with a *standard* Fisher analysis, where we evaluate the Fisher matrix of Equation (5) and derive optimal constraints using Equation (3). In this analysis, we will perform a series of sanity check to verify the robustness and validity of the computation, such as:

1. We check that the condition number^6^ 
^6^
The condition number is defined as the ratio between the maximum and the minimum eigenvalue of a given matrix. of the covariance matrix is well under 10^7^. Larger values can lead to numerical instabilities when computing the inverse of the covariance matrix.
2. We conservatively remove any frequency beyond *k*_Ny_, the Nyquist frequency of the grid.
3. We check the numerical convergence of the covariance and the derivatives by checking the change in the constraints when using a subset of the simulations. (see Figure 7).

#### 2.1.2. Fisher Analysis from Quijote Simulations

In this paper, the different Fisher computations are carried out using the Quijote Suite, which is especially designed for this purpose. We consider six cosmological parameters, {Ω_*m*_, Ω_*b*_, *h*, *n*_*s*_, *σ*_8_, *M*_*ν*_} (see Villaescusa-Navarro et al. 2020, for the choice of cosmological models). In particular, we use:

1. A set of 15,000 simulations with the same fiducial cosmology, closely matching the latest constraints by Planck (Aghanim et al. 2020), to estimate the covariance matrix.
2. A pair of 500 simulations ran with one parameter both slightly smaller and larger than the fiducial value to estimate the partial derivatives of the statistic with respect to the parameters {Ω_*m*_, Ω_*b*_, *h*, *n*_*s*_, *σ*_8_}. To compute the partial derivative of the statistic with respect to *M*_*ν*_, we instead use four sets of 500 simulations ran with *M*_*ν*_ = 0.0, 0.1, 0.2, and 0.4 eV neutrinos.

The *M*_*ν*_ = 0.0 eV simulations have the same parameters as the fiducial simulations, but they have been generated from Zeldovich initial conditions as in the massive neutrino simulations. The value of the parameters for all of the simulations employed can be found in Table 3. We refer the reader to Villaescusa-Navarro et al. (2020) for further details on the Quijote simulations.

**Figure 1.**
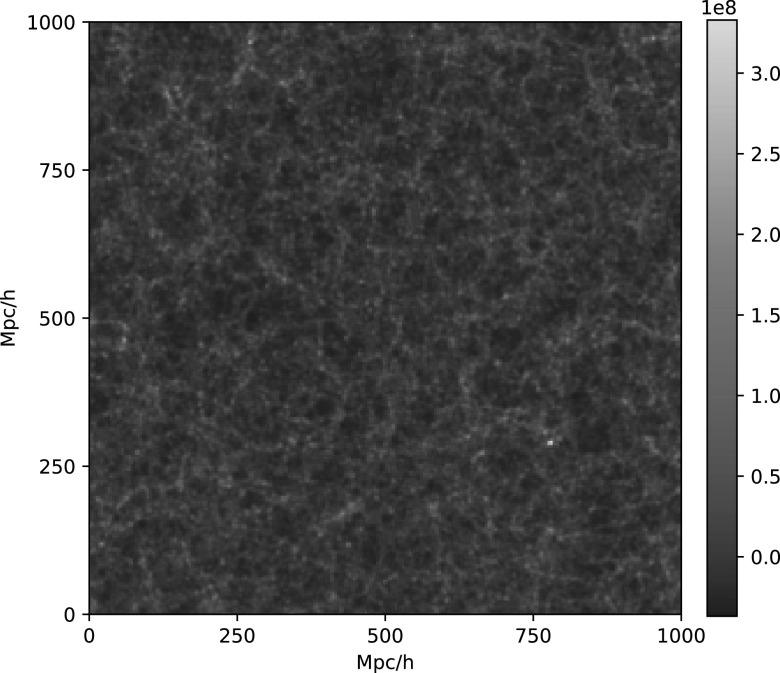
This figure shows an example of the two-dimensional density field from a Quijote simulation. The slice has dimensions of $1000\times 1000\times 250\,{({h}^{-1}\mathrm{Mpc})}^{3}$. As can be seen, these fields are non-Gaussian, and therefore the power spectrum cannot characterize all of its statistical properties.

In this work we focus our attention on summary statistics of the three-dimensional matter density field (see Figure 1 as an example). In future work, we plan to carry out this exercise for summary statistics of the halo and galaxy density fields.

### 2.2. Probing the High-dimensional Non-Gaussianity of the Statistic Distributions

Probing the Gaussianity (normality) of a probabilistic variable can be done via many tests in one dimension. For instance, a combination of the kurtosis and skewness yields a simple but efficient and fast descriptor for the non-Gaussianity (D’Agostino 1971), the Kolmogorov–Smirnov (K-S) test (Karson 1968) can evaluate the goodness-of-fit between empirical and expected cumulative distribution functions (CDFs), and the Shapiro–Wilk test (Shapiro & Wilk 1965) is another efficient test to reject a null hypothesis about Gaussianity.

However, the task becomes more complex and challenging in higher dimensions. In this work, we will perform two tests, one to identify and quantify non-Gaussian pairs, and another to quantifying whether the sharpness of the sampling distribution is reproduced by the Gaussian assumption.

#### 2.2.1. Pairwise Gaussianity Test

For some applications, it may be interesting to quantify the Gaussianity of the different dimensions of an statistic. To identify the terms exhibiting non-Gaussianity, we use a simplified version of the test proposed in Sellentin & Heavens (2017). The steps, nearly identical to Sellentin & Heavens (2017) are as follows:

1. Start with *N* samples of a *d*-dimensional statistic, ${\boldsymbol{S}}\in {{ \mathcal R }}^{(N,d)}$, where the sample mean has been subtracted, ∑_*b*_ *S*_*bi*_ = 0. (The index “b” labels the individual samples: *b* ∈ [0, *N* − 1]).
2. Compute the covariance:
  \begin{eqnarray*}{\boldsymbol{C}}=\displaystyle \frac{{{\boldsymbol{S}}}^{{\boldsymbol{T}}}{\boldsymbol{S}}}{N-d-2}\end{eqnarray*}
  and check its convergence.^7^ 
^7^
Convergence here is checked by the percent level convergence of the covariance when using 80% of the simulations. However, the convergence of the covariance does not guarantee the convergence of its inverse or any derived quantities. As we will see, we use mock data to overcome these difficulties. Note that the denominator includes the Hartlap factor (Hartlap et al. 2006).^8^ 
^8^
If we were to omit this factor, the mean of *t*_*b*_ (defined in the next item) would be away from the expected mean, *d*.
3. For all (*i*, *j*) such that 0 ≤ *i*, *j* < *d* and *i* ≠ *j*, get the two eigenvectors ***v***_(*i*,*j*)_, ***w***_(*i*,*j*)_ of the subcovariance matrix
  \begin{eqnarray*}\left(\begin{array}{cc}{C}_{{ii}} &amp; {C}_{{ij}}\\ {C}_{{ji}} &amp; {C}_{{jj}}\end{array}\right)\end{eqnarray*}
4. For all 0 ≤ *b* < *N* and all pairs (*i*, *j*), calculate:
  \begin{eqnarray*}\begin{array}{rcl}{x}_{{bij}} &amp; = &amp; {{\boldsymbol{v}}}_{(i,j)}\cdot ({S}_{{bi}},{S}_{{bj}})\\ {y}_{{bij}} &amp; = &amp; {{\boldsymbol{w}}}_{(i,j)}\cdot ({S}_{{bi}},{S}_{{bj}})\end{array}\end{eqnarray*}
  Now, if ***S*** are samples from a multivariate Gaussian, for each (*i*, *j*),
  \begin{eqnarray*}\begin{array}{l}{z}_{{bij}}={x}_{{bij}}+{y}_{{bij}}\end{array}\end{eqnarray*}
  should be samples drawn from a Gaussian as well.
5. Perform a kurtosis–skewness test (D’Agostino 1971) on *z*_*bij*_ for all (*i*, *j*) along *b* and construct the matrix:
  \begin{eqnarray*}\begin{array}{l}{R}_{{ij}}={s}_{{ij}}^{2}+{k}_{{ij}}^{2}\end{array}\end{eqnarray*}
  where s is the *z*-score from the skewness test, and k is the *z*-score from the kurtosis test, both along the sampling dimension. *s* is defined in Equation (13) in D’Agostino & Belanger (1990) while *k* is defined in Equation (19) of D’Agostino & Belanger (1990).

We will refer to this test as the pairwise Gaussianity test. The *p*-value for the test in Step 5 can also be of interest, but this is prone to numerical error and stochastic convergence, so we rather choose to run many calibrations using the covariance obtained in Step 2. We draw samples from a multivariate Gaussian having the covariance estimated in Step 2 and repeat the Gaussianity test with these samples. We perform the same tests above with this mock data. We denote the mean of *R*_*ij*_ over different mocks as ${\mu }_{{ij}}^{\mathrm{cal}}$ and the standard deviation of *R*_*ij*_ over different mocks as ${\sigma }_{{ij}}^{\mathrm{cal}}$. Note that “cal” stands for “calibration.”

#### 2.2.2. Quantifying the Overall Non-Gaussianity

The second test we use to quantify the level of non-Gaussianity of an statistic evaluates how well a multivariate Gaussian approximates the shape of the sampling distribution around the fiducial parameters. In general, this test works well when there are enough samples to obtain a converged estimate of the covariance matrix. Our test for an *s*-sigma confidence level is described below. The index *b* always runs over the different samples while *i*, *j* runs over the dimensions of the statistics:

1. Start with N samples of a *d*-dimensional statistic, ${\boldsymbol{S}}\in {{ \mathcal R }}^{(N,d)}$, where the sample mean has been subtracted, ∑_*b*_ *S*_*bi*_ = 0.
2. Divide ***S*** into two sets of *N*/2 samples. We denote the first set as ${\boldsymbol{A}}\in {{ \mathcal R }}^{(N/2,d)}$ and the second set as${\boldsymbol{B}}\in {{ \mathcal R }}^{(N/2,d)}$.
3. Compute the covariance using only ***A***:
  \begin{eqnarray*}{\boldsymbol{C}}=\displaystyle \frac{{{\boldsymbol{A}}}^{{\boldsymbol{T}}}{\boldsymbol{A}}}{N/2-d-2}\end{eqnarray*}
  and check the convergence of the matrix elements by using smaller (<*N*/2) number of samples.
4. Evaluate *t*_*b*_ = *B*_*bi*_ *C*_*ij*_ *B*_*bj*_ (no sum on b). The square root of this quantity is also called the Mahalanobis distance.
5. If the statistic distribution is Gaussian, the *t*_*b*_ values are expected to follow the *χ* ^2^-distribution for *d* degrees of freedom.
6. Use the K-S test (Karson 1968) of these *t*_*b*_ values and the *χ* ^2^-distribution of degree of freedom *d*. We get the test statistic:
  \begin{eqnarray*}{s}_{\mathrm{KS}}={\sup }_{x}| {\mathrm{CDF}}_{{t}_{b}}(x)-{\mathrm{CDF}}_{{\chi }_{d}^{2}}(x)| ,\end{eqnarray*}
  where ${\mathrm{CDF}}_{{t}_{b}}$ is the empirical CDF from the *t*_*b*_ samples and ${\mathrm{CDF}}_{{\chi }_{d}^{2}}$ is the CDF of the *χ* ^2^-distribution of degree of freedom *d*. Note that these CDFs are one-dimensional.
7. Repeat with some mock samples drawn from a Gaussian with the covariance obtained in Step 3. The test passes if the test statistic, *s*_KS_, is within an *s*-sigma interval from the Gaussian mock. In this work, we use *s* = 3 and *s* = 5.

We note that different metrics can be used to evaluate the distribution differences in Step 6. We tested out some options including the Kullback–Leibler divergence and the Earth mover’s distance, but found them to be more sensitive to the outlier samples at the tail of the distribution. We call this test the *χ* ^2^ distributional test.

With the two Gaussianity tests described above, we aim at identifying two signatures of a non-Gaussian sampling distribution: (1) when pairs of coefficients shows a highly non-Gaussian relation, and (2) when the overall sampling distribution’s peak’s sharpness differs from the Gaussian one. We will use these two tests to quantify, and remove, the non-Gaussianities of different statistics of the LSS.

### 2.3. Removing the Non-Gaussian Dimensions

Based on the above analysis, we propose a scheme to iteratively eliminate the non-Gaussian components of a given statistic, keeping a subset that passes our Gaussianity tests at some confidence level. The procedure is as follows:

1. Compute *R*_*ij*_, ${\mu }_{{ij}}^{\mathrm{cal}}$ and ${\sigma }_{{ij}}^{\mathrm{cal}}$ for all (*i*, *j*) based on Equation (7).
2. Perform the pairwise Gaussianity test:
  (a) Compute the matrix of *z*-scores *Z*_*ij*_ where
    \begin{eqnarray*}\begin{array}{l}{Z}_{{ij}}=({R}_{{ij}}-{\mu }_{{ij}}^{\mathrm{cal}})/{\sigma }_{{ij}}^{\mathrm{cal}};\end{array}\end{eqnarray*}
    this is the metric we choose to define how non-Gaussian a component is.
  (b) In order to remove the maximally non-Gaussian component, remove the row containing the maximal matrix element of *Z*_*ij*_. Since we would get two rows, we remove the row in which the sum of the *Z*_*ij*_ along the row is bigger.
  (c) Repeat (b) until all *z*-scores lay within an *s*-sigma interval.
3. Separately, perform the *χ* ^2^ distributional test:
  (a) Compute *z*_*i*_ = ∑_*j*_ *Z*_*ij*_.
  (b) Eliminate dimensions sorted by decreasing value of *z*_*i*_ until the remaining statistic passes the *χ* ^2^ distributional test within an *s*-sigma interval.
4. The remaining statistics are the dimensions surviving both tests.

We will refer to “*Gaussianize* a given statistic” when we apply to it the above procedure. It is however important to emphasize that this does not mean that we take a non-Gaussian statistic and make it Gaussian, but instead that we attempt to remove its non-Gaussian components. Thus, this procedure will naturally remove information from the statistic.

## 3. Examples with the Power Spectrum and Its Variations

We now quantify how the constraints on the value of the cosmological parameters, as derived by a Fisher matrix computation, depend on the non-Gaussianity of the considered statistic. For this, we use the power spectrum and two toy statistics that are constructed from it.

### 3.1. Statistical Probes

We start by describing the power spectrum and the two toy statistics we build from it.

#### 3.1.1. The Power Spectrum (Pk)

The power spectrum characterizes the amplitude of Fourier modes for different wavenumbers. For a homogeneous and isotropic random field, *δ*(***x***), one can define the (isotropic) power spectrum as

\begin{eqnarray*}\langle \tilde{\delta }{({\boldsymbol{k}}){\tilde{\delta }}^{* }({\boldsymbol{k}}^{\prime} )\rangle =(2\pi )}^{3}P(k){\delta }_{D}^{3}({\boldsymbol{k}}-{\boldsymbol{k}}^{\prime} )\end{eqnarray*}

where the brackets indicate an ensemble average, *δ*(***k***) is the Fourier transform of *δ*(***x***), and ${\delta }_{D}^{3}$ is a Dirac delta. Being an isotropic estimator, it depends only on the norm *k* of ***k***, the only nonvanishing configurations being for ${\boldsymbol{k}}={\boldsymbol{k}}^{\prime} $. The power spectrum, as a probe of the LSS, has the advantage of being directly interpretable and closely related to theoretical predictions.

For an isotropic and homogeneous Gaussian random field, the power spectrum contains all of the information about the underlying process. Indeed, all of the odd higher-order correlation functions vanish, and the even correlation functions can be expressed as functions of the power spectrum.

It is worth mentioning that the power spectrum of a nonlinear transformation of the density field has been shown to be a useful statistic for cosmology. For instance, the power spectrum of the log of the density field (Neyrinck et al. 2009) and the clipped power spectrum (Simpson et al. 2011, 2013) are examples of statistics that bring information from high-order correlation functions back to the power spectrum due to the nonlinear field level transformation.

**Figure 2.**
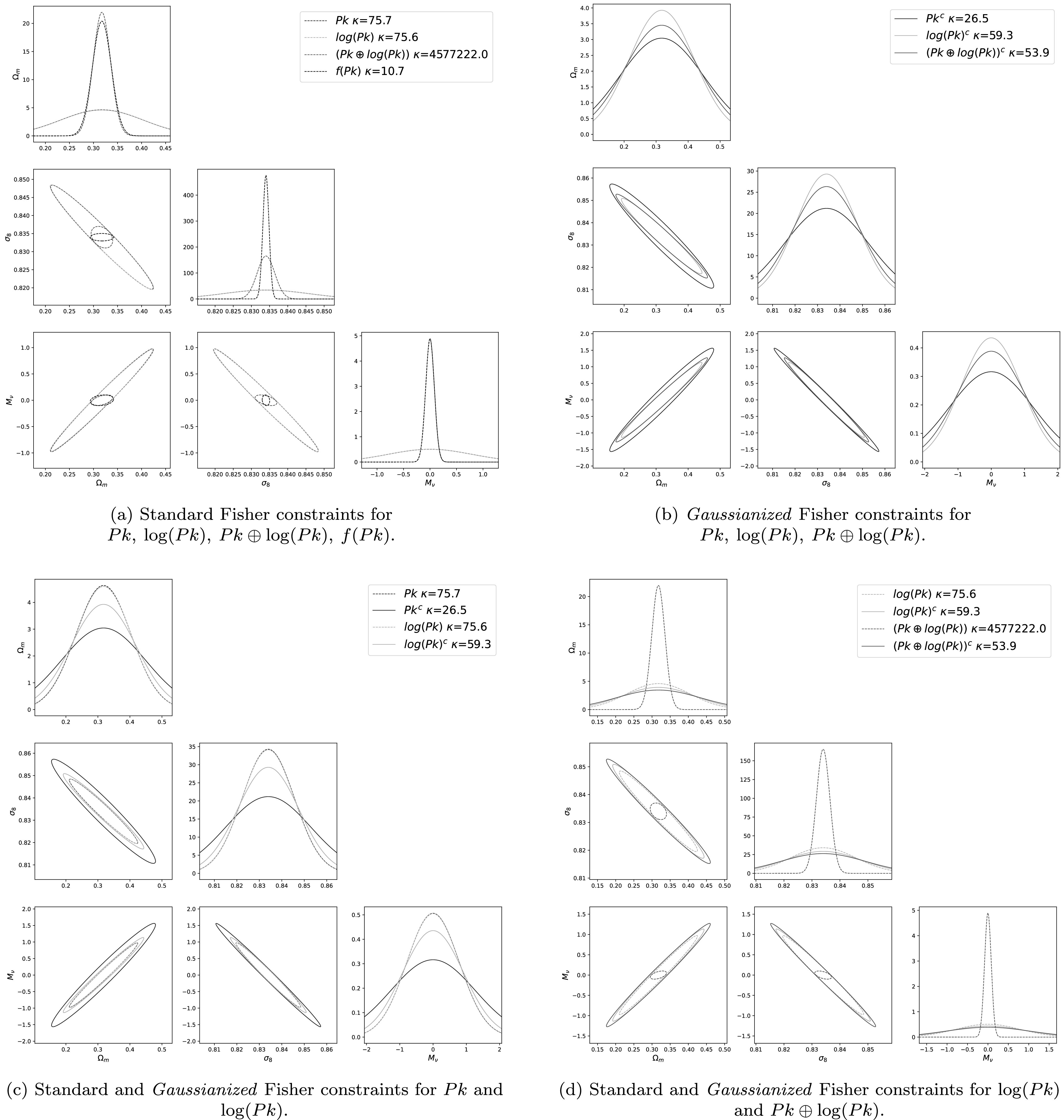
We have used the Fisher matrix formalism to quantify how well a given statistic can constrain the value of the cosmological parameters. To avoid each plot being too small, we only show the joint constraint for the three parameters Ω_*m*_, *σ*_8_, and *M*_*ν*_, while the constraints are marginalized over all six parameters. Panel (a) shows the results for $\mathrm{Pk},\mathrm{log}(\mathrm{Pk}),\mathrm{Pk}\oplus \mathrm{log}(\mathrm{Pk}),{\text{}}f(\mathrm{Pk})$, while panel (b) shows the same for the *Gaussianized* equivalent (i.e., the statistic obtained after removing the non-Gaussian components as explained in Section 2.3). All *Gaussianized* statistics are plotted in solid lines. Panel (c) has the standard and *Gaussianized* constraints for Pk and $\mathrm{log}(\mathrm{Pk})$ together, and panel (d) has the standard and *Gaussianized* constraints for $\mathrm{log}(\mathrm{Pk})$ and $\mathrm{Pk}\oplus \mathrm{log}(\mathrm{Pk})$ together. (The ellipses in panels (c) and (d) already appear in panels (a) and (b).) *κ* denotes the value of the condition number. As can be seen in panel (a), Pk ⊕ logPk and *f*(Pk) achieves tighter constraints on the value of the parameters than Pk and logPk (they achieve similar constraints), which should not be possible. Their *Gaussianized* version achieves constraints much more similar, which can be seen in panel (d). We note however that for *f*(Pk), we were not able to keep enough Gaussian dimensions to obtain reliable Fisher constraints. This exercise shows the importance of quantifying and avoiding using non-Gaussian statistics using traditional Fisher matrix calculations. The full corner plot is in Figures 8 and 9.

We have performed a standard Fisher matrix analysis using the power spectrum, and we show the results as dotted blue lines in Figure 2. We find that the results pass all standard tests: reasonable conditional number and convergence for covariance and derivatives.

#### $\mathrm{Pk}\oplus \mathrm{log}(\mathrm{Pk})$

3.1.2.

We will illustrate the problem of performing Fisher matrix analysis using non-Gaussian statistics by constructing a toy statistic whose sampling distribution is not Gaussian. We consider the statistics defined by the concatenation of the power spectrum, Pk, and the log of the power spectrum, $\mathrm{log}(\mathrm{Pk})$. We denote this statistics as $\mathrm{Pk}\oplus \mathrm{log}(\mathrm{Pk})$.

With dotted lines in Figure 2, we show the derived constraints on the value of the cosmological parameters from a standard FIM for Pk, $\mathrm{log}(\mathrm{Pk})$, and $\mathrm{Pk}\oplus \mathrm{log}(\mathrm{Pk})$. As can be seen, the constraints from the $\mathrm{Pk}\oplus \mathrm{log}(\mathrm{Pk})$ are tighter than those from Pk and $\mathrm{log}(\mathrm{Pk})$ (while these two are very similar). This is physically not possible, since we are just performing a local transformation of the power spectrum, which cannot add additional information to the existing one from the power spectrum.

One *might* think that this behavior may be only happening because Pk and logPk are very correlated, and that computing properly their cross-covariance will get the correct results. However, this is not what we found since our standard Fisher analysis passes all traditional tests to determine the robustness of the results.

#### 3.1.3. Arbitrary Transformation of the Pk: *f*(Pk)

We now show another example of an statistic derived from the power spectrum that can give rise to unrealistically tight constraints on the value of the cosmological parameters.

We build the summary statistic, which we call *f*(Pk), as follows. We optimized a multilayer perceptron (MLP) network that takes as input the power spectrum and outputs a nonlinear function of it. We minimize a loss function that represents the parameter constraints derived from a standard FIM. Specifically, we use an MLP with two hidden layers with 32 neurons, which transforms the 78-dimensional power spectrum into a 20-dimensional statistic. This approach is similar to that of Charnock et al. (2018). We apply the ReLU activation function (Glorot et al. 2011) to the output of each hidden layer. Let the parameters of the network be ***λ***, then we optimize for

\begin{eqnarray*}\lambda ^{\prime} ={\mathrm{argmax}}_{{\boldsymbol{\lambda }}}\ { \mathcal L }({\boldsymbol{\lambda }}),\end{eqnarray*}

where

\begin{eqnarray*}{ \mathcal L }({\boldsymbol{\lambda }})={{ \mathcal L }}_{{\mathrm{\Delta }}\theta }({\boldsymbol{\lambda }})+{{ \mathcal L }}_{\mathrm{NG}}({\boldsymbol{\lambda }})+{{ \mathcal L }}_{\mathrm{Cond}}({\boldsymbol{\lambda }}).\end{eqnarray*}

${{ \mathcal L }}_{{\mathrm{\Delta }}\theta }({\boldsymbol{\lambda }})$ is the loss term decreasing the marginalized parameter constraints. It is implemented as the sum of the squares of the ratio of the new constraint to the constraint given by Pk. ${{ \mathcal L }}_{\mathrm{NG}}({\boldsymbol{\lambda }})$ is the loss term maintaining the statistic to be dimensionally Gaussian. It is simply (Skewness)^2^+(kurtosis − 3)^2^. ${{ \mathcal L }}_{\mathrm{Cond}}({\boldsymbol{\lambda }})$ is just the condition number of the covariance when using this statistic. See Appendix B.2 for further details on the loss function and its different terms.

Then our statistic becomes ${\text{}}f(\mathrm{Pk})=\mathrm{MLP}({Pk},\lambda ^{\prime} )$. We show the parameter constraints, derived from the FIM in dotted lines in Figure 2. As in the case of Pk ⊕ logPk, *f*(Pk) achieves higher accuracy on the cosmological parameter than the power spectrum. This is physically not possible as both statistics are related by a transformation that does not contain cosmological information.

### 3.2. Non-Gaussianity Tests

**Figure 3.**
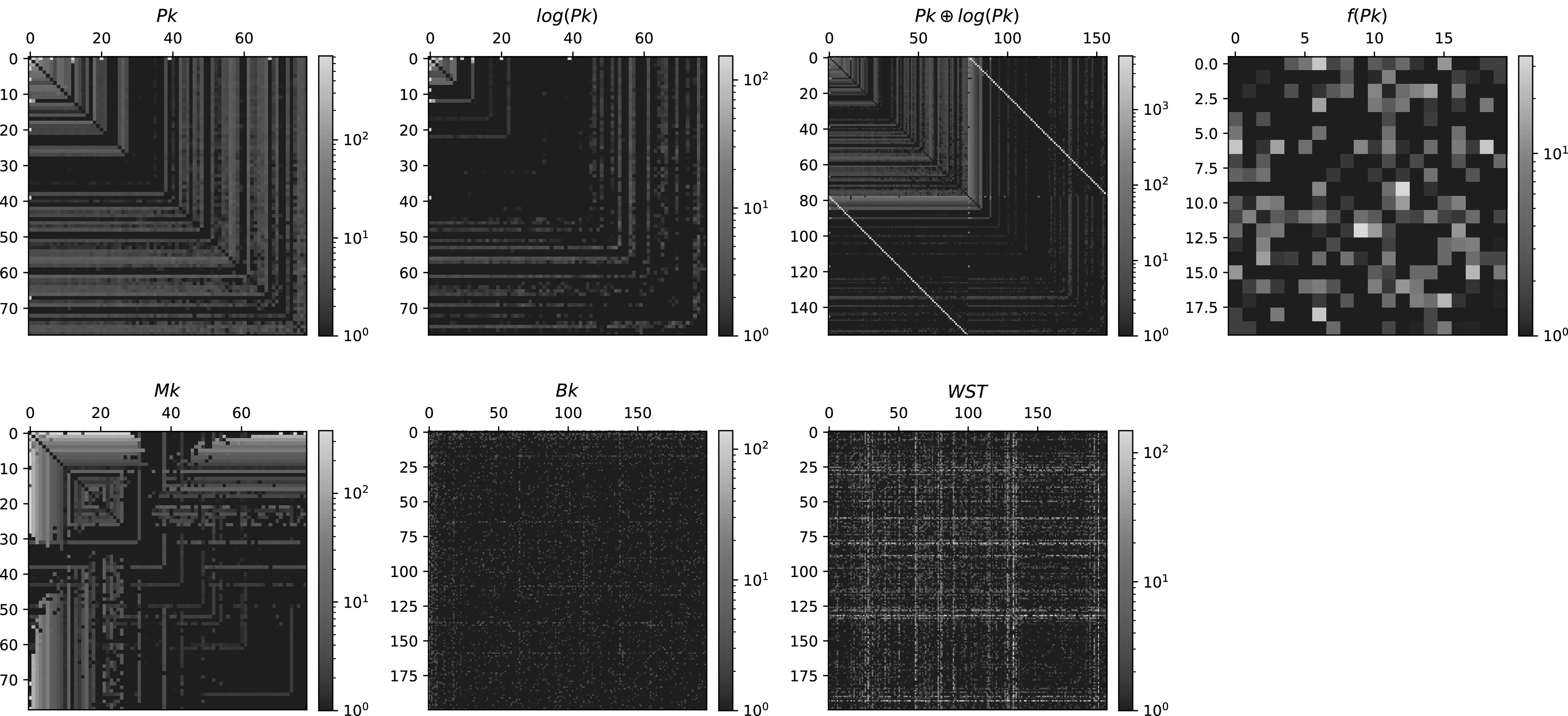
We have performed the pairwise non-Gaussianity test on a set of different statistics: Pk, $\mathrm{log}(\mathrm{Pk})$, $\mathrm{Pk}\oplus \mathrm{log}(\mathrm{Pk})$, *f*(Pk), Mk, Bk, and WST (from top left to bottom right). The color in each pixel indicates the *z*-scores, *Z*_*ij*_, defined in Equation (8). Higher values indicate larger deviations from Gaussianity. We find different patterns in the pairwise non-Gaussianity matrices. Note that Pk, $\mathrm{log}(\mathrm{Pk})$, and Mk are ordered such that the large scales (small *k*) come first. The bright bands around the 80th element of $\mathrm{Pk}\oplus \mathrm{log}(\mathrm{Pk})$ are pairs between the large scales of $\mathrm{log}(\mathrm{Pk})$ and all scales of Pk. The bispectrum, Bk, and wavelet scattering transform, WST, are reduced to 200 dimensions for the ease of analysis. This test can help us identifying and removing non-Gaussian components of a given statistic.

To investigate whether the results above are due to their sampling distribution not being Gaussian, we perform a pairwise Gaussianity test on Pk, logPk, Pk ⊕ logPk, and *f*(Pk) and show the results in the upper row of Figure 3.

For the power spectrum, we find nonnegligible non-Gaussianities at the largest scales. This is expected, since on large scales, there are few modes, and the power spectrum is not expected to follow a Gaussian distribution. This observation is somewhat similar to the one in Sellentin & Heavens (2017) for the weak lensing power spectrum. We also find some non-Gaussianities on small scales. However, we suspect this is due to numerical artifacts when calculating the power spectrum.

For the logarithm of the power spectrum, we find significantly lower non-Gaussianities, although we observe some on large scales. In this case, since the power spectrum spans several orders of magnitude, we believe that a logarithmic transform could in part reduce the effect of outliers on the covariance. For $\mathrm{Pk}\oplus \mathrm{log}(\mathrm{Pk})$, we observe that the non-Gaussianity between a dimension of Pk and the corresponding dimension of $\mathrm{log}(\mathrm{Pk})$ is clearly revealed by the pairwise Gaussianity test. For *f*(Pk), we observe some pairs with nonnegligible values of the *z*-score (Equation (8)).

**Figure 4.**
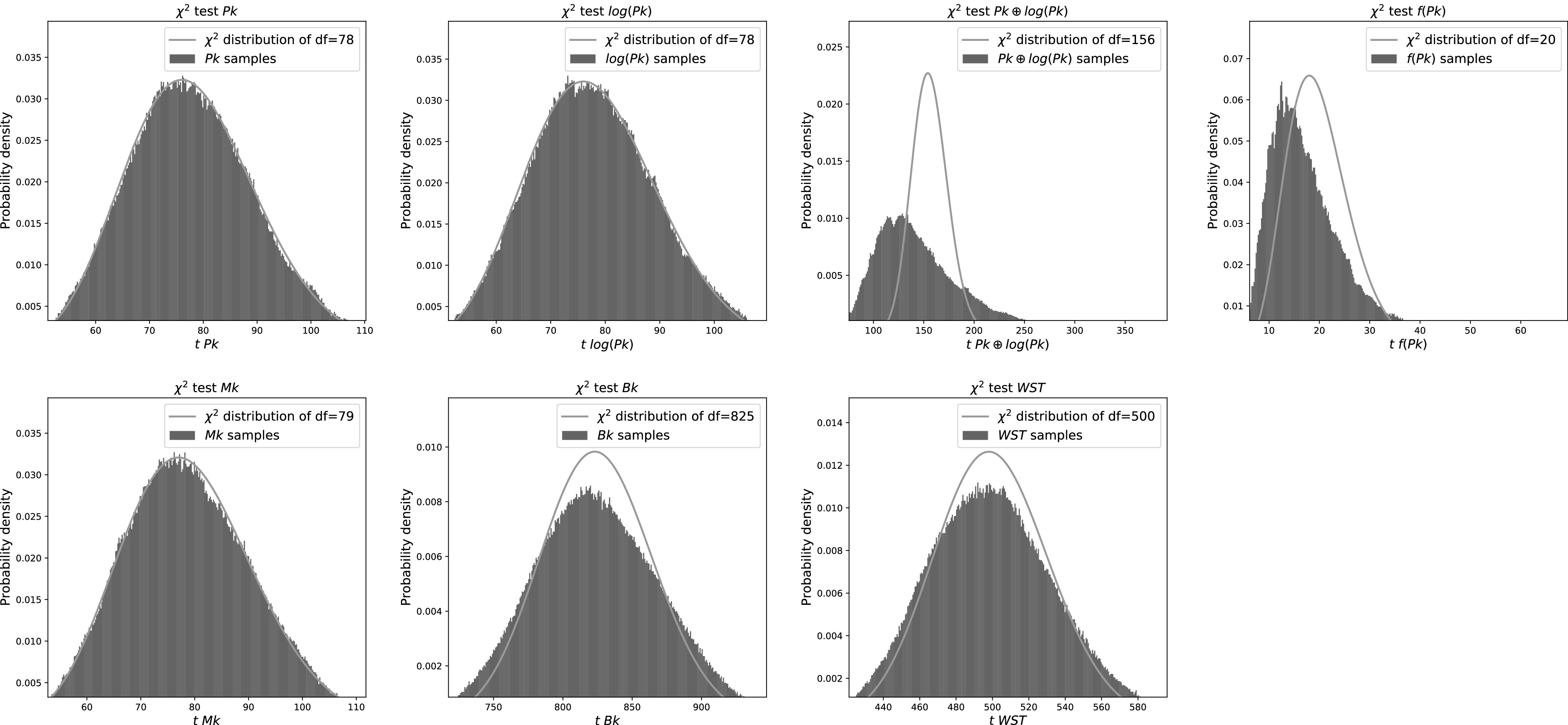
Results of the *χ* ^2^ distributional test performed on the seven statistics considered in this work: $\mathrm{Pk},\mathrm{log}(\mathrm{Pk}),\mathrm{Pk}\oplus \mathrm{log}(\mathrm{Pk}),{\text{}}f(\mathrm{Pk}),\mathrm{Mk},\mathrm{Bk},\mathrm{WST}$. As can be seen, this can help us in identifying the statistics that deviate from Gaussianity. In this case, $\mathrm{Pk}\oplus \mathrm{log}(\mathrm{Pk}),{\text{}}f(\mathrm{Pk}),\mathrm{Bk},\mathrm{WST}$ exhibit different levels of non-Gaussianity in their sampling distributions. The results of this same test after removing the non-Gaussian components are in Figures 12 and 13.

We also perform the *χ* ^2^ distributional test and show the results in the first row of Figure 4. While the CDF of *t*-values of Pk and $\mathrm{log}(\mathrm{Pk})$ shows a negligible amount of deviation from the expected *χ* ^2^ distribution, for $\mathrm{Pk}\oplus \mathrm{log}(\mathrm{Pk})$ and *f*(Pk) we find substantial deviation from the expected distribution. *f*(Pk), which was constrained to be dimension-wise Gaussian, turned out to be highly non-Gaussian and does not pass the *χ* ^2^ test. It is interesting to see that the pairwise non-Gaussianity test did not reveal these non-Gaussianities as well as it did for other probes. The reason probably lies in the way we constructed the statistic. The output of a neural network is derived from dense linear operations and nonlinearities. Thus, its output coefficients can be expected to have correlations involving many terms compared to other probes, which usually maintain some separation between the regions of Fourier plane that are probed. Even if we do not see many pairwise non-Gaussianities, it is likely that higher (>2) dimensions are correlated in a complex and non-Gaussian manner.

The above tests indicate that the results from the standard Fisher matrix calculation for the $\mathrm{Pk}\oplus \mathrm{log}\ \mathrm{Pk}$ and *f*(Pk) may not be valid since these statistics exhibit significant level of non-Gaussianities.

### 3.3. Corrected Fisher Analysis

We now *Gaussianize* the statistics using the procedure described in Section 2.3 and show the results of the FIM analysis with solid lines in panels (b)–(d) of Figure 2. We refer the reader to Tables 1 and 2 for more quantitative details.

**Table 1:** Standard and Corrected Parameter Constraints

	$\tfrac{{\mathrm{\Delta }}{{\mathrm{\Omega }}}_{m}}{{{\mathrm{\Omega }}}_{m}}$	$\tfrac{{\mathrm{\Delta }}{{\mathrm{\Omega }}}_{m}}{}{{\mathrm{\Omega }}}_{m}^{c}$	$\tfrac{{\mathrm{\Delta }}{{\mathrm{\Omega }}}_{b}}{{{\mathrm{\Omega }}}_{b}}$	$\tfrac{{\mathrm{\Delta }}{{\mathrm{\Omega }}}_{b}}{}{{\mathrm{\Omega }}}_{b}^{c}$	$\tfrac{{\mathrm{\Delta }}h}{h}$	${\tfrac{{\mathrm{\Delta }}h}{h}}^{c}$	$\tfrac{{\mathrm{\Delta }}{n}_{s}}{{n}_{s}}$	$\tfrac{{\mathrm{\Delta }}{n}_{s}}{}{n}_{s}^{c}$	$\tfrac{{\mathrm{\Delta }}{\sigma }_{8}}{{\sigma }_{8}}$	$\tfrac{{\mathrm{\Delta }}{\sigma }_{8}}{}{\sigma }_{8}^{c}$	Δ*M*_*ν*_ [*eV*]	${\mathrm{\Delta }}{M}_{\nu }^{c}\ [{eV}]$
Pk	0.271	0.433	0.752	1.109	0.683	1.038	0.463	0.73	0.014	0.023	0.789	1.273
*δ*[%]		2.9		1.4		1.7		1.7		1.7		1.0

$\mathrm{log}(\mathrm{Pk})$	0.273	0.319	0.758	0.851	0.689	0.784	0.467	0.54	0.014	0.016	0.786	0.912
*δ*[%]		1.1		0.7		0.9		1.0		0.4		0.5

$\mathrm{Pk}\oplus \mathrm{log}(\mathrm{Pk})$	0.057	0.35	0.245	0.934	0.19	0.853	0.094	0.589	0.003	0.018	0.082	0.987
*δ*[%]		8.2		6.6		6.8		7.2		5.2		5.9

*f*(Pk)	0.062	NA	0.216	NA	0.11	NA	0.055	NA	0.001	NA	0.082	NA
*δ*[%]		NA		NA		NA		NA		NA		NA

Mk	0.042	0.083	0.212	0.39	0.147	0.275	0.05	0.101	0.002	0.005	0.017	0.025
*δ*[%]		8.9		7.8		9.8		4.3		7.1		3.5

Bk	0.099	0.209	0.321	0.598	0.27	0.518	0.166	0.326	0.009	0.014	0.276	0.755
*δ*[%]		3.3		3.7		3.5		3.7		10.2		3.7

WST	0.087	0.129	0.404	0.591	0.259	0.372	0.056	0.086	0.002	0.003	0.058	0.111
*δ*[%]		6.5		2.8		3.6		2.5		4.1		10.4

**Note.** For each parameter and statistic, we describe the standard and 3*σ* corrected marginalized fractional constraints. The *δ* is the percentage error on the reported ratios of changes when using different Gaussian mocks. The neutrino mass has a fiducial value of *M*_*ν*_ = 0, so we write down the raw constraint. “NA” represents “not applicable” and is the case where the Gaussianity test leaves fewer than six dimensions.

**Table 2:** Parameter Constraint Change Ratio

	$\tfrac{{\mathrm{\Delta }}{{\mathrm{\Omega }}}_{m}^{c3}}{{\mathrm{\Delta }}{{\mathrm{\Omega }}}_{m}}$	$\tfrac{{\mathrm{\Delta }}{{\mathrm{\Omega }}}_{m}^{c5}}{{\mathrm{\Delta }}{{\mathrm{\Omega }}}_{m}}$	$\tfrac{{\mathrm{\Delta }}{{\mathrm{\Omega }}}_{b}^{c3}}{{\mathrm{\Delta }}{{\mathrm{\Omega }}}_{b}}$	$\tfrac{{\mathrm{\Delta }}{{\mathrm{\Omega }}}_{b}^{c5}}{{\mathrm{\Delta }}{{\mathrm{\Omega }}}_{b}}$	$\tfrac{{\mathrm{\Delta }}{h}^{c3}}{{\mathrm{\Delta }}h}$	$\tfrac{{\mathrm{\Delta }}{h}^{c5}}{{\mathrm{\Delta }}h}$	$\tfrac{{\mathrm{\Delta }}{n}_{s}^{c3}}{{\mathrm{\Delta }}{n}_{s}}$	$\tfrac{{\mathrm{\Delta }}{n}_{s}^{c5}}{{\mathrm{\Delta }}{n}_{s}}$	$\tfrac{{\mathrm{\Delta }}{\sigma }_{8}^{c3}}{{\mathrm{\Delta }}{\sigma }_{8}}$	$\tfrac{{\mathrm{\Delta }}{\sigma }_{8}^{c5}}{{\mathrm{\Delta }}{\sigma }_{8}}$	$\tfrac{{\mathrm{\Delta }}{M}_{\nu }^{c3}}{{\mathrm{\Delta }}{M}_{\nu }}$	$\tfrac{{\mathrm{\Delta }}{M}_{\nu }^{c5}}{{\mathrm{\Delta }}{M}_{\nu }}$
Pk	1.596	1.518	1.474	1.427	1.52	1.47	1.576	1.509	1.623	1.518	1.615	1.516
*δ*[%]	2.9	1.6	1.4	1.0	1.7	1.1	1.7	0.8	1.7	3.6	1.0	2.7

$\mathrm{log}(\mathrm{Pk})$	1.165	1.137	1.122	1.101	1.137	1.113	1.156	1.13	1.16	1.145	1.16	1.143
*δ*[%]	1.1	0.4	0.7	0.4	0.9	0.4	1.0	0.4	0.4	0.3	0.5	0.3

$(\mathrm{Pk}\oplus {log}(\mathrm{Pk}))$	6.115	5.949	3.81	3.726	4.486	4.38	6.26	6.093	6.065	5.857	12.114	11.726
*δ*[%]	8.2	8.0	6.6	6.4	6.8	6.5	7.2	7.0	5.2	5.8	5.9	6.4

*f*(Pk)	NA	NA	NA	NA	NA	NA	NA	NA	NA	NA	NA	NA
*δ*[%]	NA	NA	NA	NA	NA	NA	NA	NA	NA	NA	NA	NA

Mk	1.971	1.522	1.837	1.558	1.866	1.544	2.018	1.749	2.348	1.845	1.402	1.232
*δ*[%]	8.9	4.8	7.8	5.2	9.8	4.8	4.3	3.6	7.1	3.4	3.5	1.5

Bk	2.103	1.638	1.864	1.464	1.921	1.509	1.957	1.538	1.678	1.341	2.736	2.111
*δ*[%]	3.3	1.8	3.7	0.8	3.5	1.1	3.7	1.2	10.2	1.4	3.7	1.4

WST	1.48	1.242	1.462	1.217	1.434	1.214	1.53	1.272	1.745	1.417	1.902	1.567
*δ*[%]	6.5	1.0	2.8	4.2	3.6	1.8	2.5	4.1	4.1	4.6	10.4	3.5

**Note.** For each parameter and statistic, we describe the ratio of the new constraint to the original constraints when applying a 3*σ* condition of non-Gaussianity and a 5*σ* condition. The *δ* is the percentage error on the reported ratios of changes when using different Gaussian mocks. Since the 5*σ* condition must reject fewer terms of a statistic, it is by construction more constraining than the 3*σ* condition while allowing for more non-Gaussianity. “NA” represents “not applicable” and is the case where the Gaussianity test leaves fewer than six dimensions.

Although the non-Gaussianity detected for the power spectrum seems to be mild compared to the other probes, it does affect the parameter constraints at roughly the 50% level, as we can see from Table 2. We note however that this may be due to the fact that some of the wavenumbers identified as non-Gaussian on small scales may only be due to numerical artifacts.

For $\mathrm{log}(\mathrm{Pk})$, we find that a logarithmic transform of the power spectrum is sufficient to make it more consistently Gaussian. The corrected parameter constraints are now only corrected at the 15% level. It is important to emphasize that even if $\mathrm{log}(\mathrm{Pk})$ is just a transformation of the power spectrum, and therefore it should not contain more information that the power spectrum itself, the reason why our results show that constraints from the Gaussianized $\mathrm{log}(\mathrm{Pk})$ are better than those from the Gaussianized Pk is because our procedure removes non-Gaussian information. If that would not be the case, all statistics should give the same constraints.

For $\mathrm{Pk}\oplus \mathrm{log}(\mathrm{Pk})$, we observe that the non-Gaussianity between a dimension of Pk and the corresponding dimension of $\mathrm{log}(\mathrm{Pk})$ is clearly revealed. After correcting for the non-Gaussianity, $\mathrm{Pk}\oplus \mathrm{log}(\mathrm{Pk})$ ends up having constraints similar to that of $\mathrm{log}(\mathrm{Pk})$. We conclude that the non-Gaussian correlations and the spurious constraints caused by them are successfully removed.

For *f*(Pk), and unlike other statistics, we find it difficult to get consistent results when repeating the neural network training, or when bootstrapping the mock samples in the Gaussianity tests. In general, it should be thought to be unreliable. Although our test reveals that this statistic is exploiting the Gaussian assumption of the Fisher analysis to report seemingly confident results, we have found *f*(Pk) to sometimes (depending on the neural network instance) pass our test and report promising constraints, especially for the case of the neutrino mass. We do suspect that these constraints are still contaminated by other assumptions made for a Fisher analysis, and do not signify that a function of the power spectrum can truly be more informative. We will try to reveal the cause in a future study. This observation however suggests that a spurious probe reporting seemingly confident results could be easily engineered while being difficult to validate.

**Figure 5.**
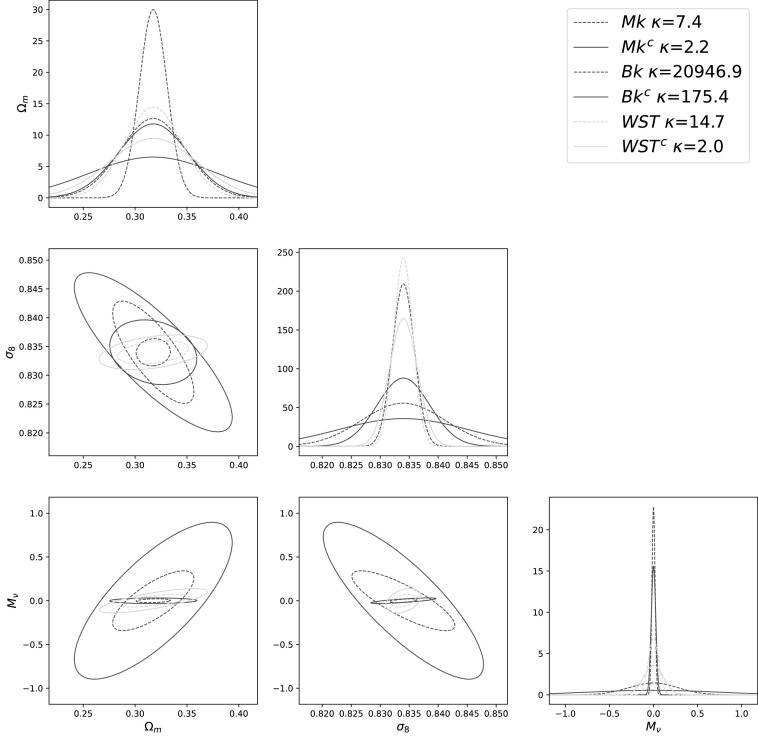
Standard (dotted lines) and *Gaussianized (solid lines)* Fisher constraints for the marked power spectrum (Mk), Bk, and WST. To avoid each plot being too small, we only show the joint constraint for the three parameters Ω_*m*_, *σ*_8_, and *M*_*ν*_, while the constraints are marginalized over all six parameters. The full corner plot is shown in Figures 10 and 11.

## 4. Application to Non-Gaussian Statistics in Cosmology

In the previous section, we illustrated the problems inherent to estimating parameter constraints using Fisher matrix calculation for statistics that exhibit some level of non-Gaussianities. In this section, we investigate the level of non-Gaussianities in statistics commonly employed to extract information not captured by the power spectrum, such as the marked power spectrum, the bispectrum, and WST. We will also study the change in the Fisher results when we Gaussianize those statistics.

### 4.1. Non-Gaussian Statistics

We now describe the different summary statistics we consider in this section. It is important to emphasize that the name of these statistics (non-Gaussian) does not arise due to their non-Gaussian distribution, but instead to the fact that they are used to study non-Gaussian density fields, where the power spectrum is not able to fully characterize its statistical properties. The sampling distribution of these statistics can still be Gaussian.

The constraints on the value of the cosmological parameters derived from a standard Fisher analysis are shown as dotted lines in Figure 5.

#### 4.1.1. The Marked Power Spectrum (Mk)

The idea behind the marked power spectrum is to assign a weight to each particle (or galaxy). That weight can be an intrinsic property of the particle/galaxy or can be related to the environment of the object.

In the cosmological context, the mark introduced by White (2016) has been studied in depth in Massara et al. (2021) and Philcox et al. (2020), especially for its ability to constrain the neutrino mass. In this work, we use the measurements from Massara et al. (2021). The mark here, first introduced in White (2016), represents an environmental property of the particle/galaxy defined as

\begin{eqnarray*}m({\boldsymbol{x}};R,p,{\delta }_{s})={\left[\displaystyle \frac{1+{\delta }_{s}}{1+{\delta }_{s}+{\delta }_{R}({\boldsymbol{x}})}\right]}^{p},\end{eqnarray*}

with parameters *R* = 10 *h* ^−1^Mpc, *p* = 2, and *δ*_*s*_ = 0.25.

#### 4.1.2. The Bispectrum (Bk)

The bispectrum is a statistic that measures correlations of closed triangles in Fourier space. For a homogeneous random field, it is defined as:

\begin{eqnarray*}\begin{array}{l}\langle \tilde{\delta }({{\boldsymbol{k}}}_{{\bf{1}}})\tilde{\delta }({{\boldsymbol{k}}}_{{\bf{2}}})\tilde{\delta }({{\boldsymbol{k}}}_{{\bf{3}}})\rangle \\ \quad =\ {\left(2\pi \right)}^{3}B(({{\boldsymbol{k}}}_{{\bf{1}}},{{\boldsymbol{k}}}_{{\bf{2}}},{{\boldsymbol{k}}}_{{\bf{3}}}){\delta }_{D}^{3}({{\boldsymbol{k}}}_{{\bf{1}}}+{{\boldsymbol{k}}}_{{\bf{2}}}+{{\boldsymbol{k}}}_{{\bf{3}}}),\end{array}\end{eqnarray*}

with the same notation as Equation (9). Note that the bispectrum, as defined above, is a scalar function with three vector arguments. However, the delta function requires ***k***_**1**_ + ***k***_**2**_ + ***k***_**3**_ = 0, i.e., the three vectors should form a triangle. Thus, the bispectrum can also be represented as *B*(*k*_1_, *k*_2_, *θ*_12_) or *B*(*k*_1_, *k*_2_, *k*_3_) assuming statistical isotropy of the field.

The bispectrum is a non-Gaussian statistic capturing interactions of different Fourier modes. In fact, the expectation value for the bispectrum vanishes for a homogenous Gaussian random field. Recently, Hahn et al. (2020) showed that the halo bispectrum is a good probe of the LSS breaking the parameter degeneracy between *σ*_8_ and the sum of the neutrino mass *M*_*ν*_.

We use our own estimator for the bispectrum, which relies on fast Fourier transforms (FFTs), similarly to other works (Sefusatti 2005; Watkinson et al. 2017). We provide further details in Appendix B.4.

#### 4.1.3. The Wavelet Scattering Transform

The WST is a set of statistics initially used in image analysis. They were first introduced in Bruna & Mallat (2013) and Mallat (2012). There are many similarities between WST and convolutional neural networks (Krizhevsky et al. 2012), since they are both built from successive applications of convolutions and nonlinearities. However, in the WST formalism, the convolutional kernels are a set of fixed wavelets instead of being optimized for the data, while the nonlinearities are a complex modulus.

Wavelets are spatially localized oscillatory functions, which probe specific frequencies and orientations. Having a set of *N*_*f*_ such wavelets that sample the whole Fourier space below the Nyquist frequency, the wavelet transform of a field *I*(***x***) is built by convolving it with these wavelets. This generates *N*_*f*_ fields, which are bandpass filtered versions of the original field on the frequencies probed by each wavelet. The WST is then built with successive application of these wavelet convolutions and nonlinear modulus operations, allowing us to characterize the interaction between different frequency components of the field (Mallat 2012). Following recent works on the WST, we restrict ourselves to a two-layer WST. Recently, the WST became a statistic of interest in astrophysical applications (Allys et al. 2019; Cheng et al. 2020; Regaldo-Saint Blancard et al. 2020; Cheng & Ménard 2021a, 2021b; Saydjari et al.^2021^).

In the present paper, to allow for a direct comparison to other three-dimensional statistics, we develop a “2.5-dimensional” WST, where instead of using fully three-dimensional wavelets, we treat the line-of-sight (LOS) direction specially. We dissect the *xy*-Fourier plane using radial and angular wavelets as in conventional two-dimensional WST, but then we multiply each of the *xy*-wavelets by every other *z*-wavelet. Our *z*-wavelets are simply logarithmically spaced one-dimensional wavelets in the *z*-direction. Our wavelets are thus not optimized to probe spherically isotropic fields but rather for a field with the LOS-direction being special. This design of these wavelets might not be optimal as a statistic for an isotropic density field, but it is motivated by the fact that the LOS-direction is treated differently in real surveys.

In this study, we use two-dimensional wavelets with eight angular bins and eight radial bins and LOS(z) wavelets with six bins. We thus have *N*_*f*_ = (1 + 8 × 8) × 6 = 390 wavelets and standardly $2+{N}_{f}+{N}_{f}^{2}=152492$ coefficients. However, we can average over angles since we assume statistical isotropy, and we assume that a convolution of a low passed image by a high-frequency filter has negligible information Mallat (2012). With these dimensionality reductions, the final dimension of the statistic is 1052. Since our Gaussianity tests are computationally intensive for high-dimensional probes, we further reduce the dimensionality to 500 dimensions by using a principal component analysis (PCA). More details are provided in Appendix B.5.

### 4.2. Results of the Non-Gaussianity Tests

We have performed the non-Gaussianity tests described in Sections 2.2.1 and 2.2.2 to the above non-Gaussian summary statistics and show the results in the bottom rows of Figures 3 and 4. We find prominent non-Gaussian pairs in the case of the marked power spectrum on large scales, and on pairs involving large and small scales. The calculation of the mark assigned to every particle requires information from some large scale, described by the parameter *R*. Philcox et al. (2020) showed that this creates a coupling between large and small scales, which may be behind this phenomenon. On the other hand, the marked power spectrum seems to pass the *χ* ^2^ distributional test (see Figure 4).

For the bispectrum, we do not find as many highly non-Gaussian pairs as we do in Mk or the WST. However, in this case, the overall non-Gaussianity revealed by the *χ* ^2^ distributional test is significant as seen in Figure 4. To check the robustness of our estimator for the bispectrum, we repeated the analysis from the public bispectrum measurements from the Quijote suite (Appendix B.4), finding similar results. When using the *χ* ^2^ test, we find substantial non-Gaussianities for both bispectra measurements (see Figure 4). We note that the presence of non-Gaussianities in the bispectrum likelihood was already noted in Scoccimarro (2000).

For the WST, Figure 3 reveals that several principal components have non-Gaussian correlations with almost all other coefficients. At this point, it is hard to reveal whether these non-Gaussianities are caused by some small amount of coefficients or a combination of them since we apply a dimensionality reduction using the principal components (see Appendix B.5). However, a linear transformation of a (multivariate) Gaussian distributed variable is still Gaussian distributed; thus, these non-Gaussianities should exist in the original coefficients. However, we warn the reader that since the amplitude of the *z*-scores (Equation (8)) is not dramatically large, these results may be affected by some inaccuracies as in the case of the power spectrum. Figure 4 shows that the *t*-values from the WST also deviate from the expected distribution in a manner similar to the bispectrum.

### 4.3. Corrected Fisher Analysis

As we did for the power spectrum, we *Gaussianize* the above non-Gaussian statistics using the procedure described in Section 2.3. With the derived statistics, we perform a Fisher matrix analysis and show the results in solid lines in Figure 5. Table 1 contains the standard and corrected constraints while their ratio can be found in Table 2.

The marked power spectrum’s parameter constraints are affected by the correction. We find that the constraints on Ω_*m*_, Ω_*b*_, Ω_*m*_, and *σ*_8_ have roughly doubled. It is also worth noting that the constraints from a 3*σ* Gaussian threshold to a 5*σ* condition are nonnegligible for the Mk, as can be seen in Table 2. We suspect that a large portion of the non-Gaussian components in Figure 3 (a) are between these thresholds. It is interesting to see that the constraint on the neutrino mass is less affected than the other parameters and still is very promising compared to the power spectrum, at least for this analysis on the three-dimensional matter density field.

For the bispectrum, the parameter constraints are also affected, resulting in constraints roughly 100% bigger (less constraining), as we can see from Table 2. The constraint on the neutrino mass (*M*_*ν*_), which is an important motivation for the bispectrum (Hahn et al. 2020), is affected by 170%, making it only different by a 10% level from the constraints from $\mathrm{log}(\mathrm{Pk})$. One could expect a similar effect for the halo or galaxy bispectra in redshift space (see Hahn & Villaescusa-Navarro 2021; Hahn et al. 2020). The extent to which this effect appears, however, would have to be estimated explicitly, and we make no claims about it in this work.

We originally had an intuition that the WST would have high levels of non-Gaussianity similarly to the bispectrum, since the same frequency components appear in the construction of several coefficients (see Appendix B.5). However, as we can read off of Table 2, the parameter change ratios were roughly similar to those of the power spectrum, except in the case of the neutrino mass. It could be the case that our principal component selection actually removed most of the complex non-Gaussianities. Nevertheless, we find corrections roughly at the 50% level, which cannot be overlooked.

We emphasize once again that the derived constraints from the Gaussianized statistics should be seen as a very conservative bound since the procedure we use to Gaussianize a statistic removes information. A full validation of the original constraints from the Fisher matrix would require us to compare them against methods that do not throw away information.

## 5. Limitations of Gaussian Tests

In this section, we describe some of the limitations of the method and tests used to (1) identify non-Gaussianities, and (2) *Gaussianize* the statistics.

In a case where one dimension is exactly a linear combination of other dimensions, the redundancy manifests in an obvious way (e.g., a large condition number or singular covariance). However, our example of $\mathrm{Pk}\oplus \mathrm{log}(\mathrm{Pk})$ is an instructive example of a non-Gaussian sampling distribution evading this check. The case here is more pernicious—the information is redundant but in a nonlinear way, which does not appear as an extremely large condition number. Nevertheless, the pairwise test makes it rather obvious which dimensions of the sampling distribution will cause the Gaussian approximation to break down.

But, in the case of inputs derived from WST, a neural network, or some other complicated statistical probe, the issue is further complicated for two reasons, as follows:

1. The presence of nonlinear relations between its dimensions cannot be easily guessed, as it is the case for $\mathrm{Pk}\oplus \mathrm{log}(\mathrm{Pk})$, where we do suspect such a relation from construction.
2. Such a relation could be a nonlinear combination of many dimensions, which can be hard to detect by the pairwise Gaussianity test.

And thus, although our correction scheme renders the distribution of these statistic more compatible to a Gaussian approximation, we expect there to be many different ways a statistic could be non-Gaussian while evading the pairwise test. As a simple example, we point out that a three-component relation cannot be easily picked up with this test. Let us consider

\begin{eqnarray*}a\sim { \mathcal N }(0,1)\end{eqnarray*}

\begin{eqnarray*}b\sim { \mathcal N }(0,1)\end{eqnarray*}

\begin{eqnarray*}c=\displaystyle \frac{a+b+\epsilon \times {(a+b)}^{3}}{\sqrt{2}}.\end{eqnarray*}

When *ϵ* is zero, every two-dimensional joint distribution will be exactly amultivariate Gaussian while the full three-dimensional distribution will clearly not be. In fact, the covariance will be singular in this case, and this is something that can be easily spotted. However, when *ϵ* is not zero but small, the covariance will not be singular nor have a very big condition number. Every two-dimensional subdistribution will still be very close to Gaussian, and thus the pairwise non-Gaussianity test will fail to detect the severe non-Gaussianity. Extending the pairwise non-Gaussianity test to a triplet test would reveal the relation; however, this approach does not scale well with the dimensionality of the probe.

Although this toy example seems to be artificially tailored to show this effect, similar cases are expected to show up in real data. The *ϵ* = 0 case is rarely seen in real data since such an explicit linear relation is usually discovered using linear analysis. However, nearly linear relations with slight nonlinearities are expected to be a common case, even though the nonlinear components might not be of any known form, as in the example above. In general, for a *d*-dimensional statistical probe, if one can predict a single dimension of the statistic using the *d* − 1 dimensions better than what a Gaussian process could do, a hidden relation between the dimensions of the statistics should be suspected to exist.

**Figure 6.**
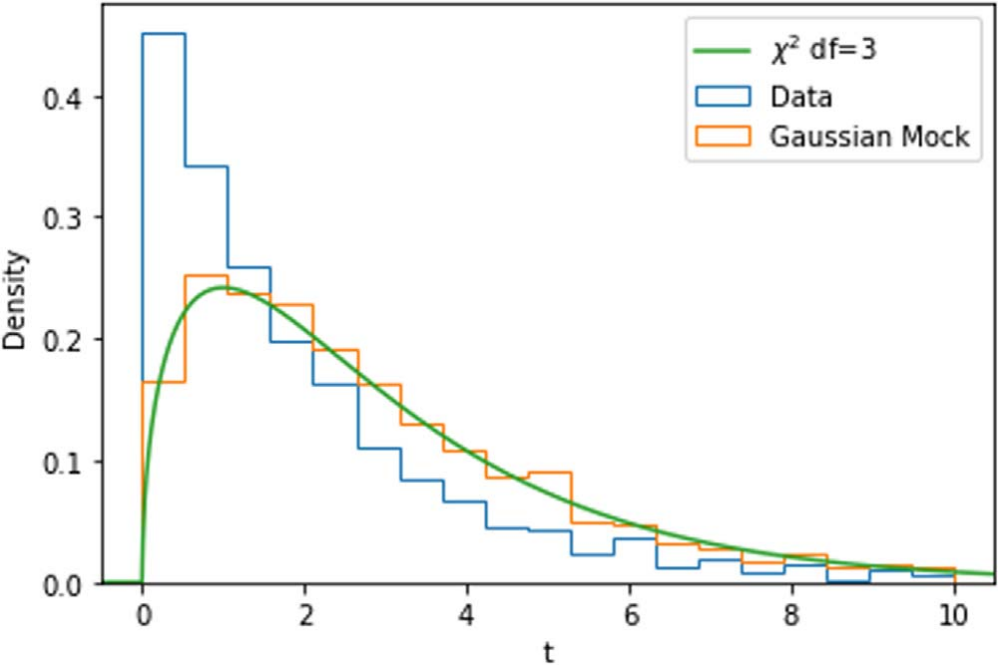
The non-Gaussianity of the joint distribution of a, b, and c in Equation (14) revealed by the *χ* ^2^ distributional test.

**Figure 7.**
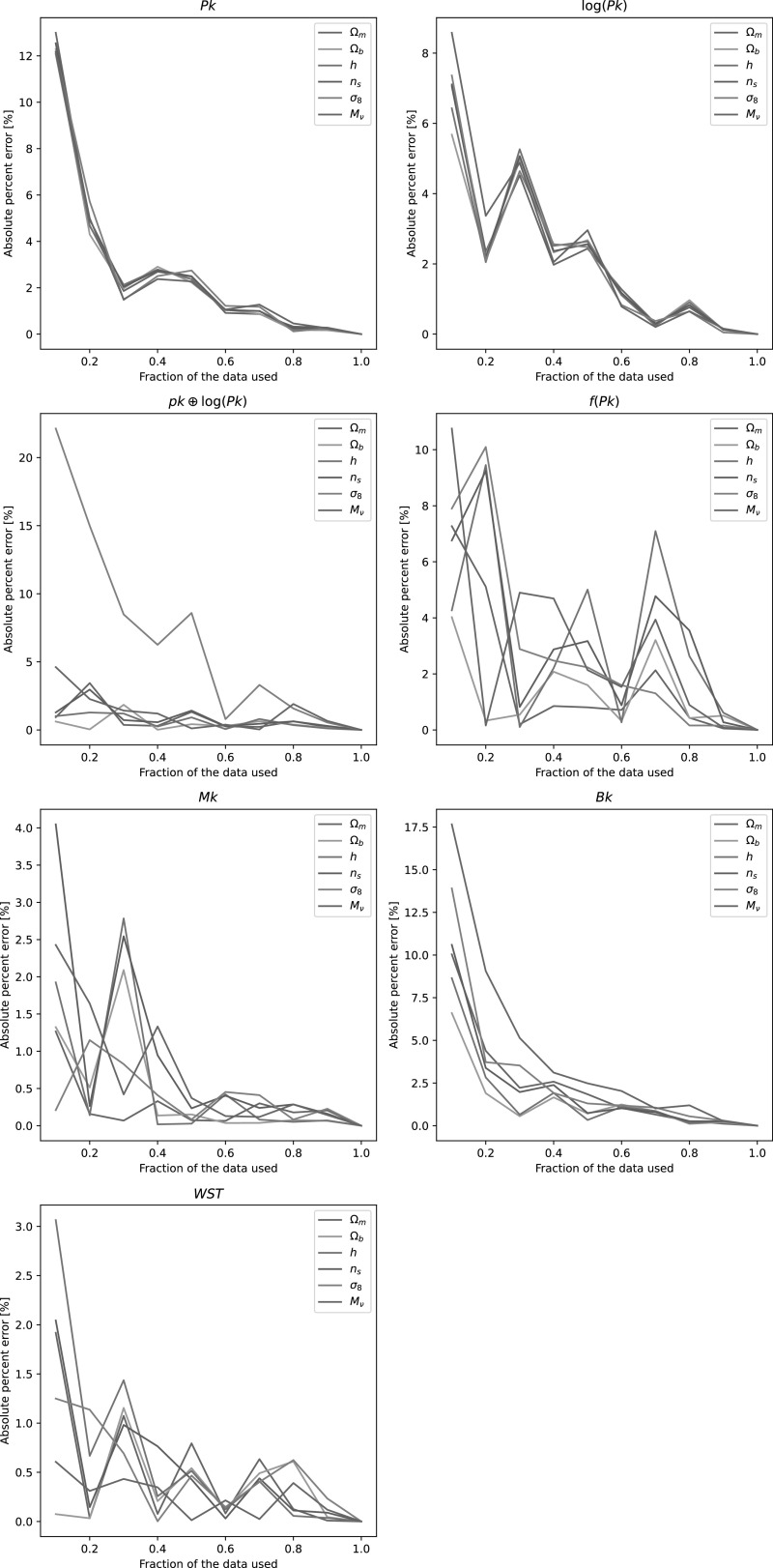
The convergence of the parameter constraints are shown. We used a subset of the fiducial and derivative simulations to repeat the whole Fisher analysis. The absolute percent error on the parameter constraints are plotted as a function of the fraction of simulations used. Using 80% of the simulations, the error for all statistics and all parameters is at the percent level.

Further elaborating on this example, the non-Gaussianity here is detected by our *χ* ^2^ distributional test, as we can see from Figure 6. This is because even though every two-dimensional subdistribution is Gaussian, the *t*-values of the samples are not consistent with a Gaussian distribution. Our *χ* ^2^ distributional test thus serves as a complementary test to the pairwise Gaussianity test.

In the statistical probes explored, our *χ* ^2^ distributional test was effective and indispensable in picking out the non-Gaussianity for *f*(Pk), Bk, and WST, but we note that the test is somewhat less sensitive than the pairwise test. In complex statistics like the bispectrum and the WST, there could be very complicated hidden relations connecting some dimensions in a complicated highly non-Gaussian manner. In this sense, our *χ* ^2^ distributional test is a good way to complement the pairwise test.

Finally, it should be noted that passing these tests should be treated as a necessary condition but not a sufficient one. There are many ways non-Gaussianities can hide in high-dimensional distributions, while we only check for the cases where (1) two dimensions of the statistic have a non-Gaussian partial distribution, and (2) where the sharpness of the Gaussian approximation of the sampling distribution is vastly different from the one computed from data.

Let us consider the example of the *f*(Pk) statistic. While this statistic cannot contain more information than the power spectrum, the results from the Fisher may be interpreted in the other direction when the Gaussian tests are passed. This clearly illustrates the limitations of the proposed tests. In general, one should thus always simultaneously check for convergence, numerical stability, and Gaussianity when performing a Fisher analysis and be as rigorous as possible. We thus highlight that the interpretability of a statistical probe has a major importance, especially when using machine-learning typed approaches, since they provide an intuition of how the joint distribution will behave.

## 6. Conclusions

The Fisher matrix formalism is commonly used in cosmology to quantify the accuracy that a given statistic can constrain the value of some cosmological parameters. This method will determine the variance of the optimal unbiased estimator for the considered statistic. However, the Fisher matrix is usually computed assuming that the statistic considered follows a multivariant Gaussian distribution.

In this work, we have considered several statistics to characterize the LSS of the universe and investigated whether their distribution is Gaussian or deviate from it. For this, we made use of two tests that will identify pairwise and global non-Gaussian distributions of the considered statistic. These tests can be employed in general and are not only designed for Fisher matrix calculations. We found non-Gaussianities in traditional statistics like the power spectrum and bispectrum but also in more recent statistics like the marked power spectrum and WST. We note that our conclusions are in agreement with previous works that have investigated this in depth (see, e.g., Hahn et al. 2019).

Next, we have applied a procedure to *Gaussianize* the statistics, which consists of identifying the non-Gaussian components of the statistic and removing them. We stress that this procedure removes non-Gaussian dimensions, rather than Gaussianizing the entire statistic. We have then performed Fisher matrix calculations with the standard and the Gaussianized statistics. We find significant corrections to the parameter constraints: (62%, 51%) for the power spectrum, (134%, 84%) for the marked power spectrum, (173%, 111%) for the bispectrum, and (90%, 56%) for the WST when the threshold to Gaussianize the statistics is set to (3*σ*, 5*σ*), respectively.

We have also shown that without imposing Gaussianity for a given statistic, one can achieve unrealistically tight constraints on the value of the parameters. We illustrated this by considering the statistics $\mathrm{Pk}\oplus \mathrm{log}(\mathrm{Pk})$ (the concatenation of the power spectrum and the logarithm of it) and *f*(Pk), which performed better than the power spectrum just by a nonlinear transformation that does not contain cosmological information.

We have also outlined the limitations of the method we use in this work, which can identify pairwise and global (around the peak) non-Gaussianities, but cannot identify more complex non-Gaussianities (e.g., higher-order interactions). It is also important to mention that we found that the Gaussianized statistics performed worse in constraining the value of the parameters. An obvious reason for this is because our method throws away the non-Gaussian information. A fairer comparison would be to develop an optimal method to Gaussianize a given statistic or to perform the inference with a method that did not rely on a Gaussian assumption, e.g., likelihood-free inference (see, e.g., Charnock et al. 2018; Alsing et al. 2019; Diaz Rivero & Dvorkin 2020; Makinen et al. 2021). Thus, the degraded constraints derived in this work from the Gaussianized statistics should be recalled as a conservative and perhaps more robust bound. This work however emphasizes the need to compare the constraints derived from the Fisher matrix with methods that do not discard the non-Gaussian information.

We note that other methods may be more efficient at Gaussianize statistics. For instance, Scoccimarro (2000) proposed the use of PCA components of the bispectrum as a way to compress the relevant information while at the same time taking advantage of the central limit theorem to Gaussianize the likelihood. We note that this strategy is similar to the one we have used for the WST, although the *χ* ^2^ test revealed the presence of non-Gaussianities. One could also calculate the correction to the distribution of the statistic as in Hall & Taylor (2022).

In general, Fisher matrix calculations are known to perform well at the 10% level. In this work, we have shown that under more conservative assumptions, the Fisher constraints can be trusted within a factor of ∼2, at least for the statistics considered in this work. The tests used in this work can thus be used to quantify the robustness of the considered statistics to Fisher matrix assumptions.

In the quest to find the best statistic to constraint the value of the cosmological parameters, it is important to keep in mind the inherent limitations of the Fisher matrix formalism. The method used in this work will allow us to complement the standard analysis with a more conservative Fisher matrix calculation. These, combined with methods like simulation-based inference can help the community identify robust statistics to retrieve cosmological information from the LSS of the universe.

We note that the Gaussianity of a given statistic not only affects the outcome of Fisher matrix calculations, but traditional analyses performed using, for instance, Markov Chain Monte Carlo methods (see, e.g., Byun et al. 2021; Philcox & Ivanov 2022) commonly assume a Gaussian likelihood. If this assumption breaks down, corrections to the inferred parameters would also be expected.

We release the code we have used to compute the power spectra, bispectra, and WST. The code can be found on GitHub^9^ 
^9^
LazyWaveletTransform codebase: https://zenodo.org/badge/latestdoi/346117283. and is archived in Zenodo and works on both CPUs and GPUs.

## Figures and Tables

**Figure 8.**
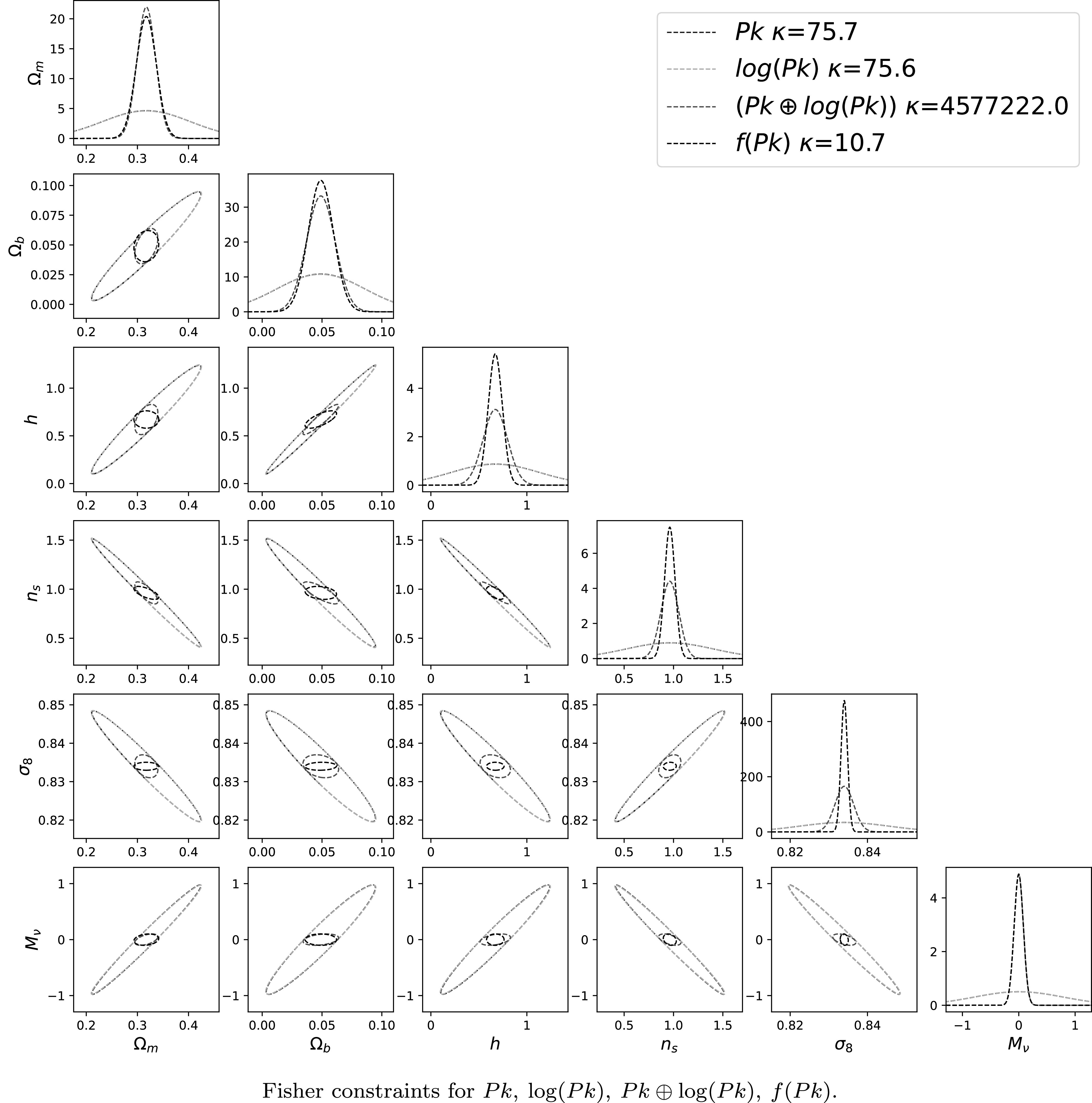
We used the Fisher matrix formalism to quantify how well a given statistic can constrain the value of the cosmological parameters. The figure shows the results for $\mathrm{Pk},\mathrm{log}(\mathrm{Pk}),\mathrm{Pk}\oplus \mathrm{log}(\mathrm{Pk}),{\text{}}f(\mathrm{Pk})$. Pk and $\mathrm{log}(\mathrm{Pk})$ almost overlap and appear as a single line. As can be seen, Pk ⊕ logPk and *f*(Pk) achieve tighter constraints on the value of the parameters than Pk and logPk (they achieve similar constraints), which is not possible.

**Figure 9.**
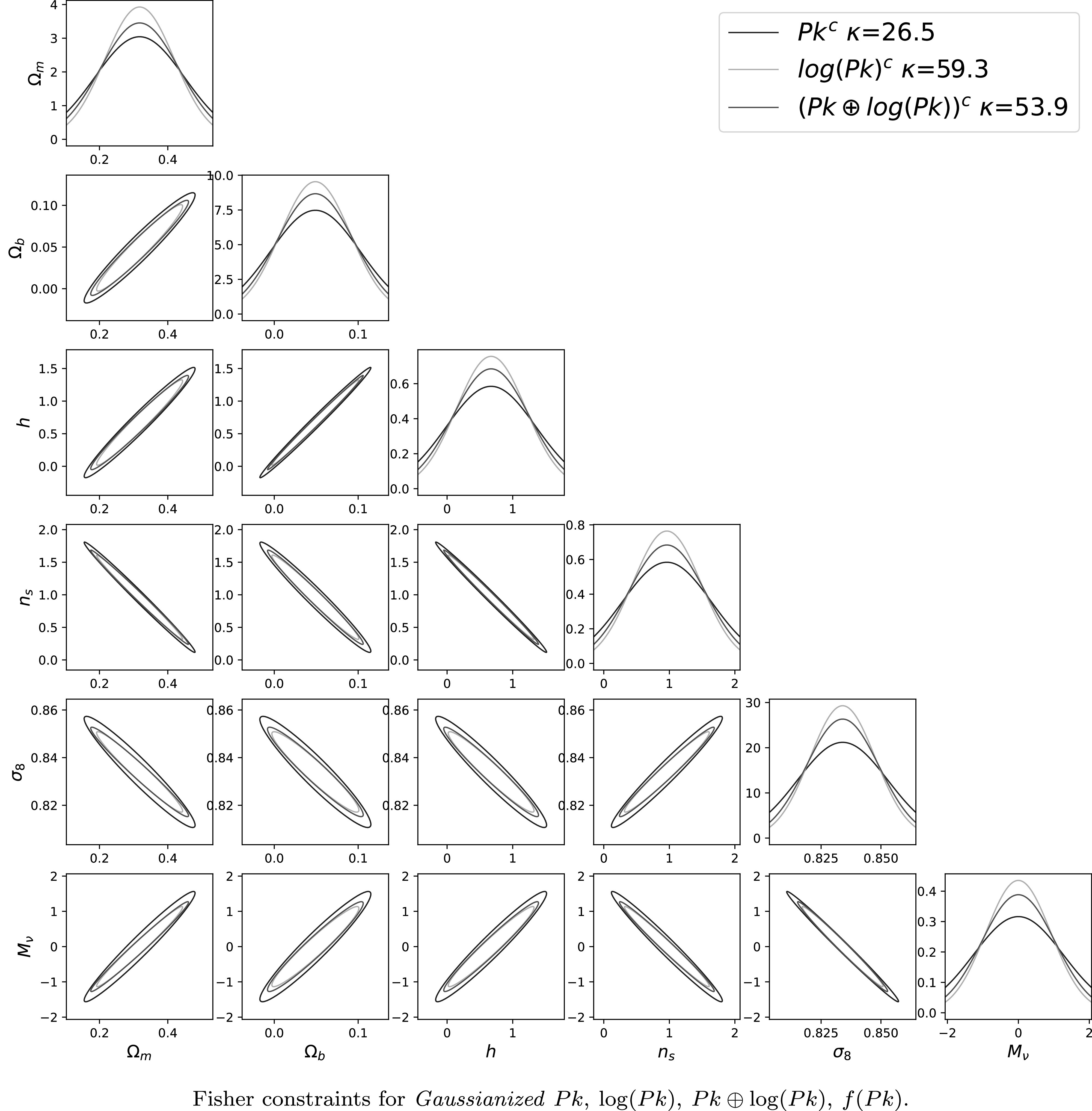
Similarly to Figure 8, we show the results for $\mathrm{Pk},\mathrm{log}(\mathrm{Pk}),\mathrm{Pk}\oplus \mathrm{log}(\mathrm{Pk})$, but for the Gaussianized equivalent (i.e., the statistic obtained after removing the non-Gaussian components as explained in Section 2.3). The *Gaussianized* $\mathrm{Pk}\oplus \mathrm{log}(\mathrm{Pk})$ achieve constraints much more similar to either Pk or logPk. We note however that for *f*(Pk) we were not able to keep enough Gaussian dimensions to obtain reliable Fisher constraints.

**Figure 10.**
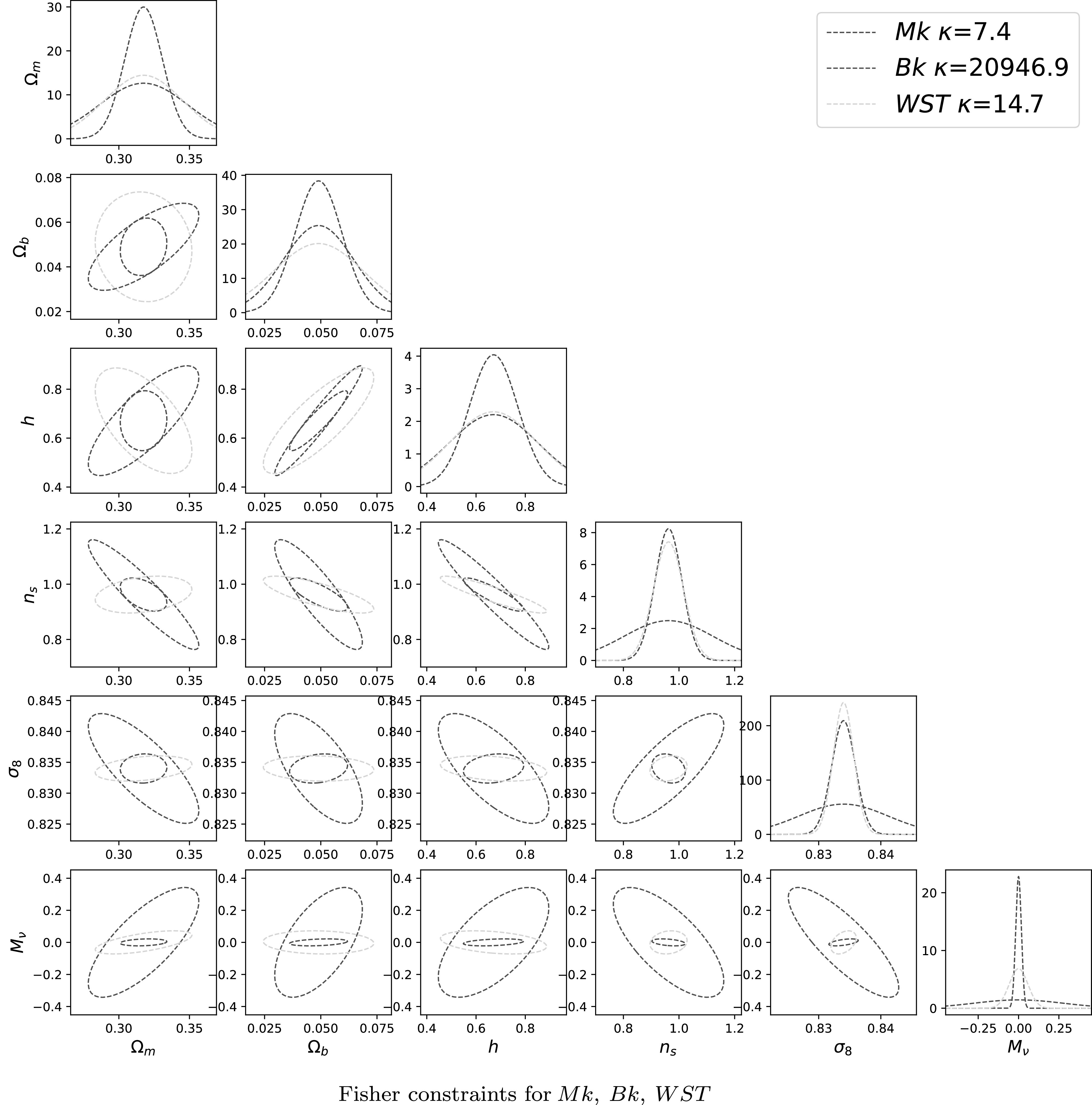
Same as Figure 8 but for the marked Mk, Bk, and WST.

**Figure 11.**
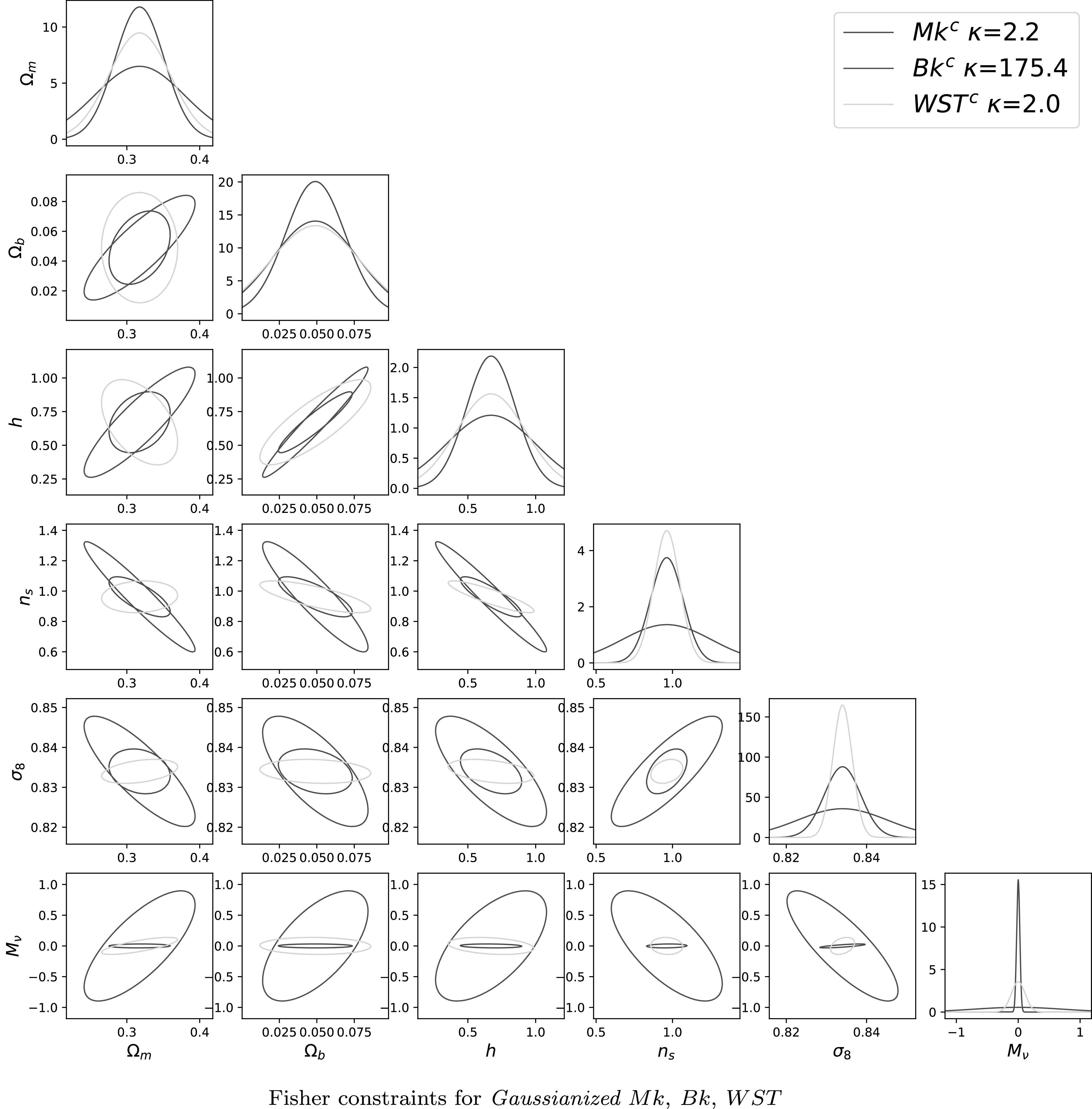
Same as Figure 9 but for the *Gaussianized* marked Mk, Bk, and WST. We see that some covariance ellipses change shape (e.g., Bk for *σ*_8_ and *h*), direction (e.g., Mk for *σ*_8_ and Ω_*b*_), and size (most ellipses).

**Figure 12.**
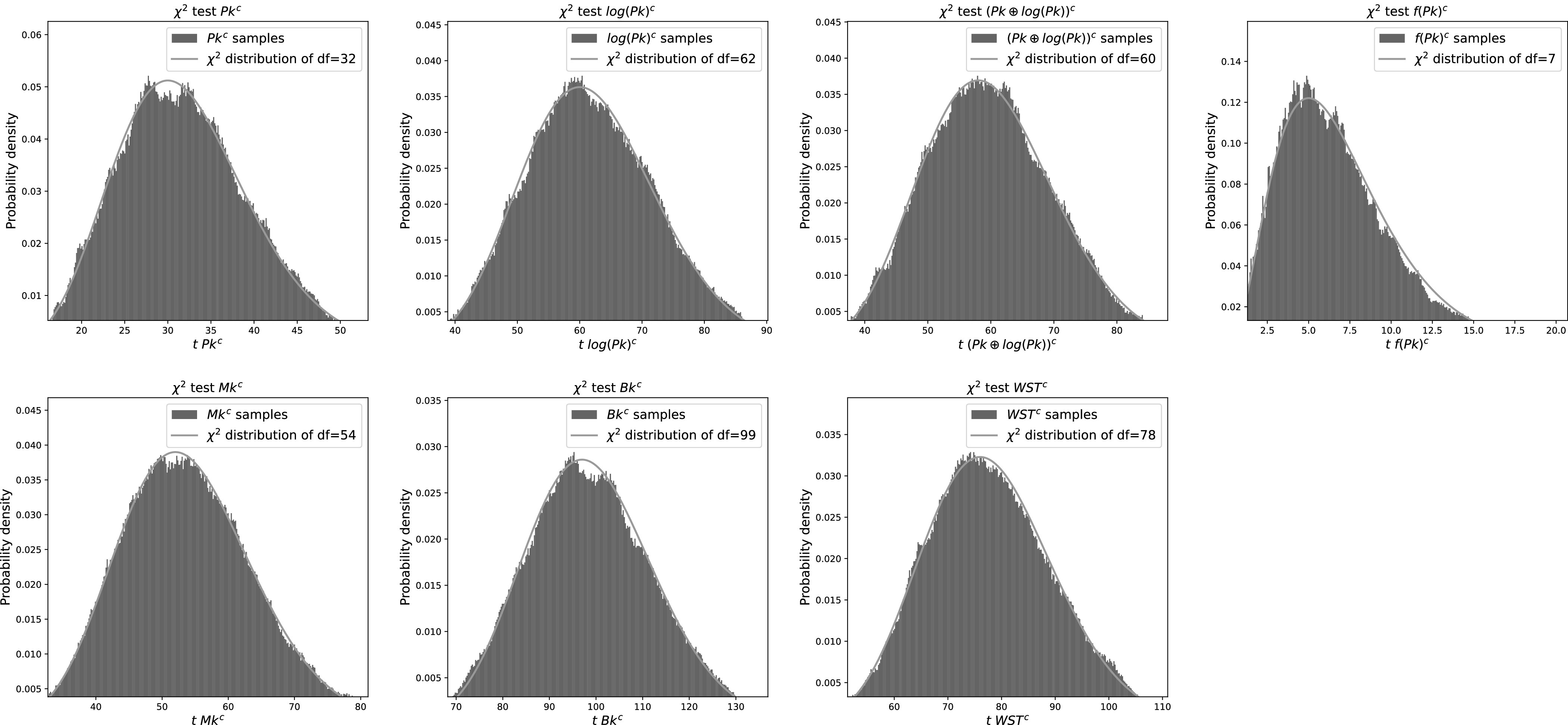
Results of the *χ* ^2^ distributional test performed on the seven statistics considered in this work: $\mathrm{Pk},\mathrm{log}(\mathrm{Pk}),\mathrm{Pk}\oplus \mathrm{log}(\mathrm{Pk}),{\text{}}f(\mathrm{Pk}),\mathrm{Mk},\mathrm{Bk},\mathrm{WST}$. Unlike Figure 4, this test was performed after removing the non-Gaussian components at the 3*σ* level.

**Figure 13.**
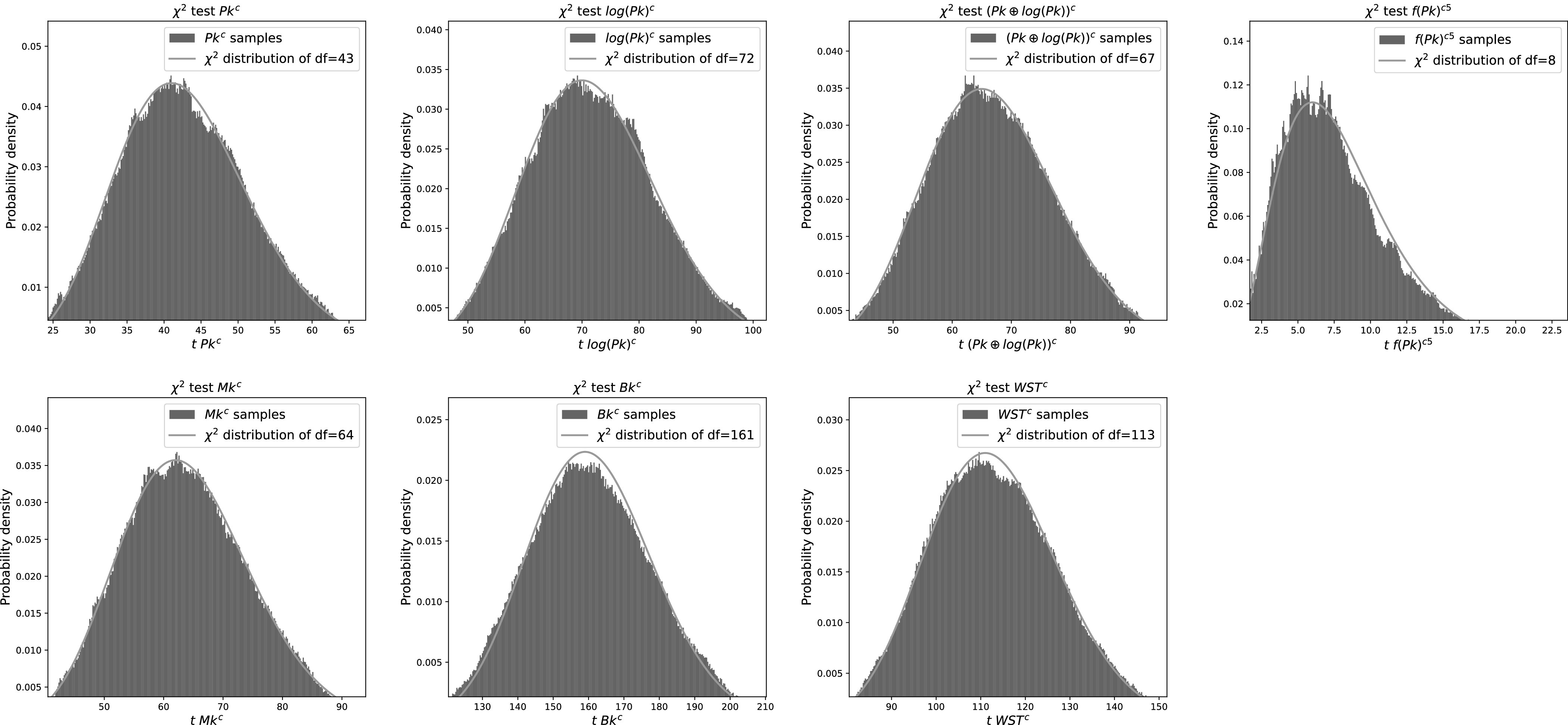
Results of the *χ* ^2^ distributional test performed on the seven statistics considered in this work: $\mathrm{Pk},\mathrm{log}(\mathrm{Pk}),\mathrm{Pk}\oplus \mathrm{log}(\mathrm{Pk}),f(\mathrm{Pk}),\mathrm{Mk},\mathrm{Bk},\mathrm{WST}$. Unlike in Figure 4, this test was performed after removing the non-Gaussian components at the 5*σ* level.

**Figure 14.**
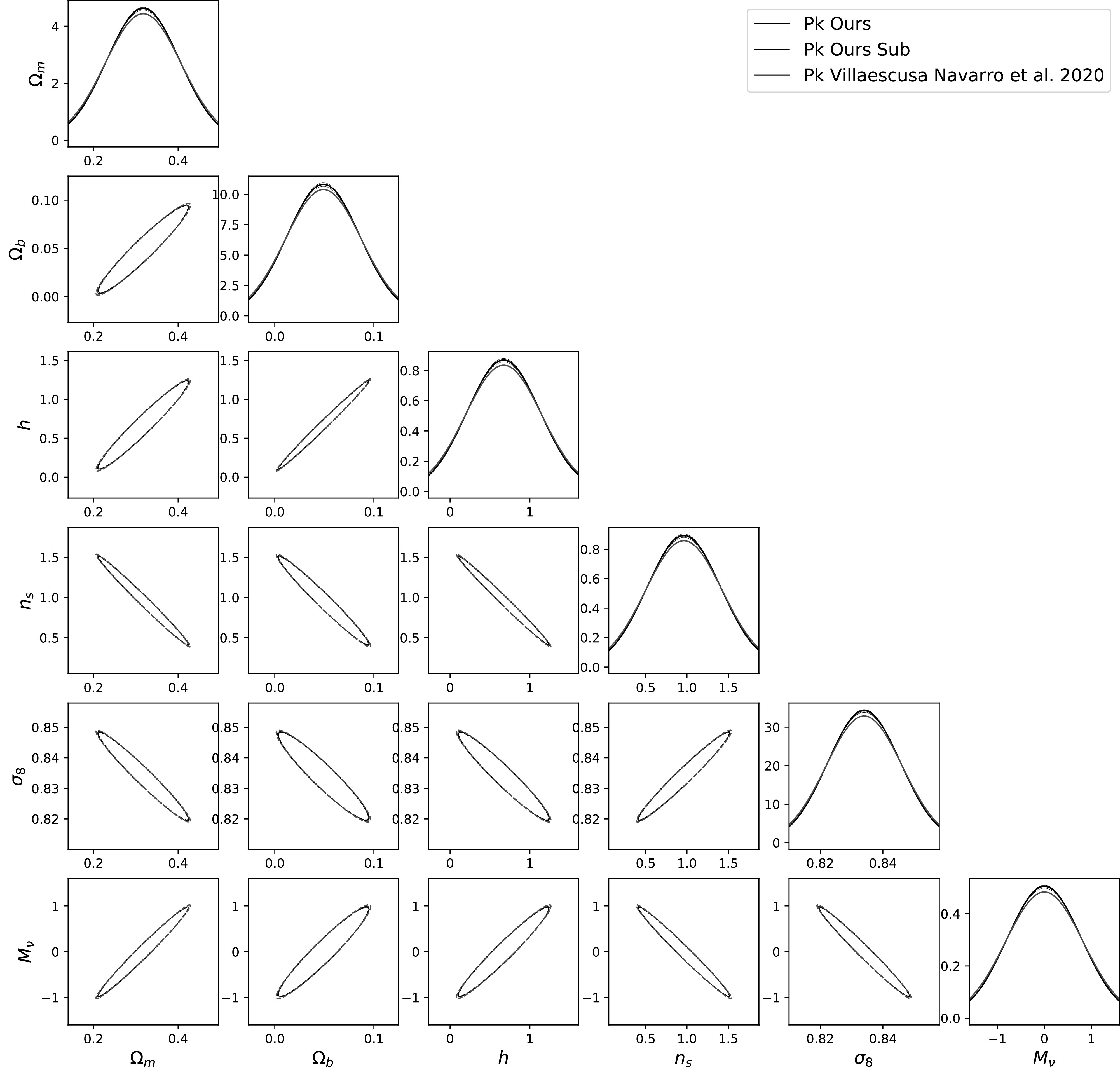
Comparison of Fisher matrix constraints from our power spectrum and the power spectrum from Villaescusa-Navarro et al. (2020).

**Figure 15.**
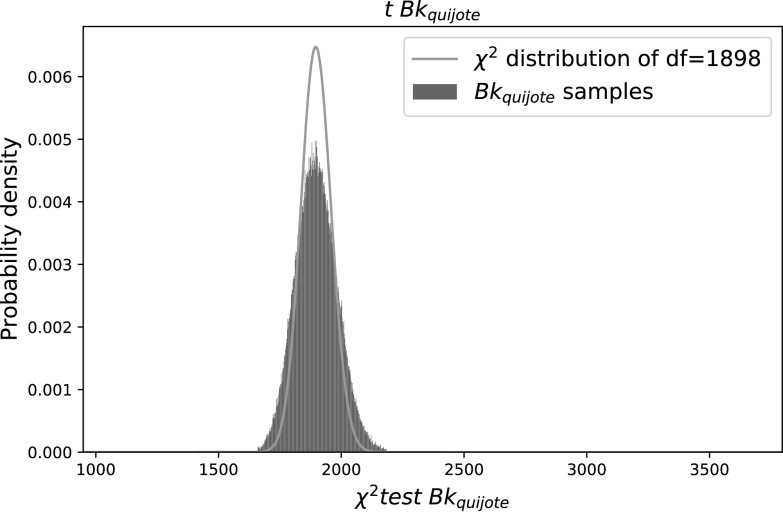
*χ* ^2^ distributional test applied to the 1898-dimensional bispectrum from Villaescusa-Navarro et al. (2020).

## Appendix A Parameters of the Simulations

Table 3 contains the characteristics of the Quijote *N*-body simulations used for the Fisher matrix calculations in this work. We refer the reader to Villaescusa-Navarro et al. (2020) for further details on the Quijote simulations.

**Table 3:** Parameters of the Simulations

Name	*N*	*L* [*h* ^−1^Mpc]	IC	Ω_*m*_	Ω_*b*_	*h*	*n*_*s*_	*σ*_8_	*M*_*ν*_[eV]
Fiducial	N	1000	2LPT	0.3175	0.049	0.6711	0.9624	0.834	0.0
${{\mathrm{\Omega }}}_{m}^{+}$	15000	1000	2LPT	**0.3275**	0.049	0.6711	0.9624	0.834	0.0
${{\mathrm{\Omega }}}_{m}^{-}$	500	1000	2LPT	**0.3075**	0.049	0.6711	0.9624	0.834	0.0
${{\mathrm{\Omega }}}_{b}^{+}$	500	1000	2LPT	0.3175	**0.051**	0.6711	0.9624	0.834	0.0
${{\mathrm{\Omega }}}_{b}^{-}$	500	1000	2LPT	0.3175	**0.047**	0.6711	0.9624	0.834	0.0
*h* ^+^	500	1000	2LPT	0.3175	0.049	**0.6911**	0.9624	0.834	0.0
*h* ^−^	500	1000	2LPT	0.3175	0.049	**0.6511**	0.9624	0.834	0.0
${n}_{s}^{+}$	500	1000	2LPT	0.3175	0.049	0.6711	**0.9824**	0.834	0.0
${n}_{s}^{-}$	500	1000	2LPT	0.3175	0.049	0.6711	**0.9424**	0.834	0.0
${\sigma }_{8}^{+}$	500	1000	2LPT	0.3175	0.049	0.6711	0.9624	**0.849**	0.0
${\sigma }_{8}^{-}$	500	1000	2LPT	0.3175	0.049	0.6711	0.9624	**0.814**	0.0
*M*_*ν*_	500	1000	Zeldovich	0.3175	0.049	0.6711	0.9624	0.834	**0.0**
${M}_{\nu }^{+}$	500	1000	Zeldovich	0.3175	0.049	0.6711	0.9624	0.834	**0.1**
${M}_{\nu }^{++}$	500	1000	Zeldovich	0.3175	0.049	0.6711	0.9624	0.834	**0.2**
${M}_{\nu }^{+++}$	500	1000	Zeldovich	0.3175	0.049	0.6711	0.9624	0.834	**0.4**

**Note.** *N* is the number of simulations, and *L* denotes the size of the box in comoving units. “IC” denotes the method of initial condition generation.

## Appendix B Details of the Statistics

### B.1. Power Spectrum

We use the well-known “FFT and bin” method to compute the power spectrum. We bin the squared amplitudes into uniform bins spaced by the frequency resolution: ${k}_{\mathrm{res}}=2\pi /L$ where *L* is the length of the box. For the sake of clarity, our bin edges are $[-0.5\ {k}_{\mathrm{res}},0.5\ {k}_{\mathrm{res}},\cdots ,\,\left(\sqrt{3}\lfloor H/2\right.\unicode{x0230B}+0.5)\ {k}_{\mathrm{res}}]$ for a grid side H. The factor of $\sqrt{3}$ comes from the three-dimensional nature of the grid. Although, as is clear from the above, we bin all of the modes resulting from an FFT, to avoid contamination from any information from ∣ ***k*** ∣ > *k*_Ny_, we only use the bins below 0.5 *k*_Ny_

### B.2. *f*(Pk)

We discuss the details of our information maximizing neural network. We use a multilayer perceptron architecture with an ReLU activation (Glorot et al. 2011). A single sample of the input power spectrum with 78 elements, ${\boldsymbol{P}}{\boldsymbol{k}}\in {{ \mathcal R }}^{78}$, is processed as follows:

\begin{eqnarray*}\begin{array}{rcl}{{\boldsymbol{x}}}_{{\bf{1}}} &amp; = &amp; ({\boldsymbol{P}}{\boldsymbol{k}}-{\boldsymbol{\mu }})\oslash {\boldsymbol{\sigma }}\\ {{\boldsymbol{x}}}_{{\bf{2}}} &amp; = &amp; \mathrm{ReLU}({{\boldsymbol{\Theta }}}_{1}{{\boldsymbol{x}}}_{{\bf{1}}}+{{\boldsymbol{b}}}_{{\bf{1}}})\\ {{\boldsymbol{x}}}_{{\bf{3}}} &amp; = &amp; \mathrm{ReLU}({{\boldsymbol{\Theta }}}_{2}{{\boldsymbol{x}}}_{{\bf{2}}}+{{\boldsymbol{b}}}_{{\bf{2}}})\\ {{\boldsymbol{x}}}_{{\bf{4}}} &amp; = &amp; {{\boldsymbol{\Theta }}}_{3}{{\boldsymbol{x}}}_{{\bf{3}}}+{{\boldsymbol{b}}}_{{\bf{3}}}\\ f &amp; := &amp; {\boldsymbol{P}}{\boldsymbol{k}}\to {{\boldsymbol{x}}}_{{\bf{4}}}\end{array}\end{eqnarray*}

where ${\boldsymbol{x}}2,{\boldsymbol{x}}3\in {{ \mathcal R }}^{32}$, ${\boldsymbol{x}}4\in {{ \mathcal R }}^{20}$, and ⊘ constitute the Hadamard division. The matrices **Θ**_*i*_ have dimensions compatible for the vector dimensions. The scaling vectors *μ*, *σ* are fixed to the fiducial mean and standard deviation. This transformation alone is a linear transform and thus does not affect the Fisher analysis up to numerical effects. However, neural networks perform optimally when the data is ${ \mathcal O }(1)$ motivating this transform.

Calling the vector of all parameters, {**Θ**_1_, **Θ**_2_, **Θ**_3_, *b*_1_, *b*_2_, *b*_3_} as ***λ***, we optimize for

\begin{eqnarray*}{ \mathcal L }({\boldsymbol{\lambda }})={{ \mathcal L }}_{{\mathrm{\Delta }}\theta }({\boldsymbol{\lambda }})+{{ \mathcal L }}_{\mathrm{NG}}({\boldsymbol{\lambda }})+{{ \mathcal L }}_{\mathrm{Cond}}({\boldsymbol{\lambda }})\end{eqnarray*}

using the Adam optimizer (Kingma & Ba 2014) until convergence. ${{ \mathcal L }}_{{\mathrm{\Delta }}\theta }({\boldsymbol{\lambda }})$ is simply defined as the sum of rational change of the marginalized parameter constraints, ${{ \mathcal L }}_{\mathrm{NG}}({\boldsymbol{\lambda }})$ quantifies the dimensionally non-Gaussianity of the samples; it is the squared sum of the normalized skew and the kurtosis. ${{ \mathcal L }}_{{Cond}}({\boldsymbol{\lambda }})$ is the condition number of the covariance matrix times a small constant, here 0.001. While the first two losses are of order unity, the condition number of the matrix is generally considered safe when under 10^8^. We discount this term to focus the network on optimizing the two first functions. To avoid any potential issues from over-fitting to these specific realizations of the simulation, we only use 70% of the simulations.

### B.3. Marked Power Spectrum

We use the mark in Equation (12), with optimal parameters *R* = 10 h^−1^Mpc, *p* = 2, and *δ*_*s*_ = 0.25. We do not calculate this but use the publicly available data from Villaescusa-Navarro et al. (2020).

### B.4. Bispectrum

Here are our choices when implementing Equation (13) with the FFT estimator (Sefusatti 2005; Watkinson et al. 2017), as follows:

1. Our computational representation of *δ*(∣ ***k*** ∣ − *k*)), or a “*k*-ring” centered at *k,* is smoothed. We use a *b*-splined kernel as the WST (see Equation (B.5)) falling off to 0 at the center of the neighboring bins. It is normalized to unity.
2. We use 16 *k*-values uniformly sampled in $\mathrm{log}(k)$ up to *k*_Ny_.
3. We use all possible triangles satisfying the triangular inequality.

We end up with 825 valid configurations.

### B.5. Reduced Wavelet Scattering Transform

We describe our three-dimensional WST and its reduction scheme. We start with wavelets similar to the Morlet wavelets in two dimensions. Then we multiply a one-dimensional wavelet in the *z*-direction. Thus we treat the *z*-axis as being different than *x* and *y* motivated by the line-of-sight (LOS)-direction in the observational scenario. One could interpret these as 2.5-dimensional wavelets having one dimension different than the two others. For NZ LOS wavelets, NR radial wavelets, and NT angular wavelets, our three-dimensional wavelets are ${\psi }_{k}^{z}\times {\psi }_{{ij}}^{{xy}}$(*x,y,z* are just naming labels) where 0 ≤ *k* < NZ, 0 ≤ *i* < NR, 0 ≤ *j* < NT, and we add the DC wavelets ${\psi }_{k}^{\mathrm{DC}}$ to keep the squared sum of the wavelets unity near the DC frequency.

Although one can use any LOS, radial, angular separations, we use radial bins and LOS bins equally spaced in logarithmic space and equally spaced angular bins:

\begin{eqnarray*}{r}_{i}=\left\{\begin{array}{l}0\ \mathrm{when}\ i< 0\\ \mathrm{pow}\left(2,1+i\ast \displaystyle \frac{{\mathrm{log}}_{2}({k}_{\mathrm{Ny}})-1}{\mathrm{NR}}\right)\ \mathrm{when}\ 0\leqslant i< \mathrm{NR}\\ {\mathrm{log}}_{2}({k}_{\mathrm{Ny}})\ \mathrm{when}\ \mathrm{NR}\leqslant i\end{array}\right.\end{eqnarray*}

\begin{eqnarray*}{\theta }_{j}=j\ast \displaystyle \frac{\pi }{\mathrm{NT}}\end{eqnarray*}

\begin{eqnarray*}{z}_{k}=\left\{\begin{array}{l}0\ \mathrm{when}\ i< 0\\ \mathrm{pow}\left(2,1+i\ast \displaystyle \frac{{\mathrm{log}}_{2}({k}_{\mathrm{Ny}})-1}{\mathrm{NZ}}\right)\ \mathrm{when}\ 0\leqslant i< \mathrm{NZ}\\ {\mathrm{log}}_{2}({k}_{\mathrm{Ny}})\ \mathrm{when}\ \mathrm{NZ}\leqslant i\end{array}\right.\end{eqnarray*}

where *k*_Ny_ is the Nyquist frequency. Note that we define the values at all *i* in order to simplify our wavelet definitions below.

We then define the *b*-spline:

\begin{eqnarray*}{b}_{0}(t)=\left\{\begin{array}{l}1\ \mathrm{when}\ {\mathrm{t}}< 0\\ 2{t}^{3}-3t+1\ \mathrm{when}\ 0\leqslant {\mathrm{t}}< 1\\ 0\ \mathrm{when}\ 1\leqslant {\mathrm{t}}\end{array}\right.\end{eqnarray*}

\begin{eqnarray*}{b}_{1}(t,{t}_{i},{t}_{f})={b}_{0}\left(\displaystyle \frac{t-{t}_{i}}{{t}_{f}-{t}_{i}}\right)\end{eqnarray*}

\begin{eqnarray*}{b}_{2}(t,{t}_{c},{t}_{l},{t}_{r})=\left\{\begin{array}{l}0\ \mathrm{when}\ t< {t}_{l}\\ {b}_{1}(t,{t}_{c},{t}_{l})\ \mathrm{when}\ {{\mathrm{t}}}_{{\mathrm{l}}}\leqslant {\mathrm{t}}< {{\mathrm{t}}}_{{\mathrm{c}}}\\ {b}_{1}(t,{t}_{c},{t}_{r})\ \mathrm{when}\ {{\mathrm{t}}}_{{\mathrm{c}}}\leqslant {\mathrm{t}}< {{\mathrm{t}}}_{{\mathrm{r}}}\\ 0\ \mathrm{when}\ {{\mathrm{t}}}_{{\mathrm{r}}}\leqslant {\mathrm{t}}\end{array}\right..\end{eqnarray*}

Our wavelets are then:

\begin{eqnarray*}\begin{array}{l}{\psi }_{{ij}}^{{xy}}(r,\theta )={b}_{2}(r,{r}_{i},{r}_{i-1},{r}_{i+1})\times {b}_{2}(\theta ,{\theta }_{i},{\theta }_{i-1},{\theta }_{i+1})\end{array}\end{eqnarray*}

\begin{eqnarray*}{\psi }_{k}^{z}(z)={b}_{2}(z,{z}_{i},{z}_{i-1},{z}_{i+1})\end{eqnarray*}

\begin{eqnarray*}{\psi }_{{ijk}}(r,\theta ,z)={\psi }_{{ij}}^{{xy}}(r,\theta )\times {\psi }_{k}^{z}(z)\end{eqnarray*}

to where we add the DC wavelets

\begin{eqnarray*}{\psi }_{k}^{{DC}}(r,\theta ,z)=\left\{\begin{array}{l}0\ \mathrm{when}\ {r}_{0}< r\\ (1-{\sum }_{i,j}{\psi }_{{ij}}^{{xy}}(r,\theta ))\times {\psi }_{k}^{z}(z)\end{array}\right..\end{eqnarray*}

We thus have NF = (1 + NR × NT) × NZ wavelets.

Indexing all wavelets as ***ψ***_*i*_ (note that we abuse the notation here to represent the function sampled at all pixels needed to match the image size), the wavelet coefficients for an input image ***I*** are defined as:

\begin{eqnarray*}\mu =\mathrm{mean}({\boldsymbol{I}})\end{eqnarray*}

\begin{eqnarray*}\sigma =\mathrm{std}({\boldsymbol{I}})\end{eqnarray*}

\begin{eqnarray*}\overrightarrow{S0}=\{\mu ,\sigma \}\end{eqnarray*}

\begin{eqnarray*}{I}^{0}=\displaystyle \frac{{\boldsymbol{I}}-\mu }{\sigma }\end{eqnarray*}

\begin{eqnarray*}{I}_{i}^{1}={{\boldsymbol{I}}}^{0} \circledast {{\boldsymbol{\psi }}}_{i}\end{eqnarray*}

\begin{eqnarray*}{\mathrm{S}}{1}_{i}=\mathrm{mean}({{\boldsymbol{I}}}_{{\boldsymbol{i}}}^{{\bf{1}}})\end{eqnarray*}

\begin{eqnarray*}{I}_{{ij}}^{2}=| {I}_{i}^{0}{| }^{2} \circledast {{\boldsymbol{\psi }}}_{j}\end{eqnarray*}

\begin{eqnarray*}S{2}_{{ij}}=\mathrm{mean}({{\mathrm{I}}}_{{ij}}^{2})\end{eqnarray*}

\begin{eqnarray*}\overrightarrow{\mathrm{WST}}=\{\overrightarrow{S0},\overrightarrow{{\mathrm{S}}1},\overrightarrow{{\mathrm{S}}2}\}.\end{eqnarray*}

The mean and std operations are ran over the image domains, all bold fonts represent images, superscripted arrows represent vectors.

The total number of WST coefficients are then 2 + NF + NF^2^. For this analysis, we discard the field mean, which should be zero for all of the overdensity fields. Due to the high dimensionality, we reduce the dimensionality by reporting the angular averaged coefficients. In detail, we report *S*1 coefficients averaged over angles. We then divide all *S*2 coefficients by the corresponding *S*1 coefficient to remove redundant information. We then only take the coefficients where the angular index and the LOS index for the first convolution and the second convolution are the same. We then take the averaged coefficient over this angle. For a Fisher analysis, it would be useful to have an even smaller dimensionality due to numerical effects and convergence. We thus report only the first 500 principal components derived from the set of coefficients from the fiducial simulations. These coefficients have strictly less information than the whole set of coefficients.

In practice, we deviate from the original WST introduced by Bruna & Mallat (2013) in two senses:

1. We use *b*-spline wavelets; these wavelets are indexed by *R* and *T* where *R* represents the radial index and *T* represents the angular index.
2. We take the absolute value squared instead of the absolute value as the nonlinear operation between convolutions.

We use *b*-spline wavelets for some motivations:

1. ${\sum }_{{ij}}{\psi }_{{ij}}^{2}+{\psi }_{\mathrm{DC}}^{2}=1$ everywhere in the Fourier disk up to $| {\boldsymbol{k}}| ={k}_{\max }$. The wavelet square sums to unity up to *k*_max_. Thus, the summed coefficients are expected to be more isotropic. Then it decays to zero from *k*_max_ to *k*_Ny_.
2. In addition to the above property, the wavelets decays to zero from $| {\boldsymbol{k}}| ={k}_{\max }$ to ∣ ***k*** ∣ = *k*_Ny_, and thus we have no contribution at all from any modes over *k*_Ny_.
3. The wavelets decays precisely to zero within a sparse region of the Fourier space. This permits a much faster computation using the bounding boxes of the wavelets.

## Appendix C Convergence of the Parameter Constraints

We used a subset of the fiducial and derivative simulations to estimate the convergence of the parameter constraints. The entire Fisher analysis was repeated using a fraction of the simulations and the results are shown in Figure 7.

## Appendix D Full-sized Corner Plots for the Fisher Constraints

We show the full 6 parameter corner plots for all standard and corrected statistics. The standard constraints for $\mathrm{Pk},\mathrm{log}(\mathrm{Pk}),\mathrm{Pk}\oplus \mathrm{log}(\mathrm{Pk}),f(\mathrm{Pk})$ are shown in Figure 8 and their corrected constraints in Figure 9. The standard constraints for $\mathrm{Mk},\mathrm{Bk},\mathrm{WST}$ are shown in 10 and their corrected constraints in Figure 11.

## Appendix E Result of the *χ* ^2^ Distributional Test on the Corrected Statistics

We have applied the *χ* ^2^ distributional test on the corrected statistics as well. As expected, the statistics corrected at the 3*σ* level and at the 5*σ* level were most consistent with the null hypothesis that the statistic is Gaussian distributed. These results are shown in Figures 12 and 13.

## Appendix F Computational Details

We make our library computing Pk, Bk, WST publicly available. Our code is available on Github.

We discuss some details we consider to accelerate the computation.

1. All of our functions are batched. Modern machines’ RAM and GPUs can easily have >10 GB of memory. To perform FFTs and array slicing in an efficient manner, we batch every function. For a two-dimensional field, one thus feeds in a (C,H,W) array, and for a three-dimensional field, one feeds in a (C,H,W,D) array.
2. When the nonlinearity applied between convolutions is the modulus squared for the WST, we do not perform the last IFFT since we can use the Plancherel theorem:
  \begin{eqnarray*}{\int }_{-\infty }^{\infty }| f(x){| }^{2}{dx}={\int }_{-\infty }^{\infty }| \hat{f}(k){| }^{2}{dk}.\end{eqnarray*}
  We can thus simply output the squared power multiplied by the wavelet in Fourier space.
3. Most of our wavelets are sparse in Fourier Space. One can use a sparse representation and extract the relevant pixels of the Fourier space image. However, since sparse operations are inherently slower than dense operations, we use a much faster alternative. We exploit the fact that our wavelets are not only sparse in Fourier space but also compactly packed in a small region. (It is important that one computationally works in the Fourier space representation where the zero frequency is in the middle of an array.). We simply precompute the rectangular bounding box of each wavelet and only operate on the pixels in the bounding box. The remaining sparcity after extracting out only the bounding box is of the order of 1.
4. Since a Fourier transform of an image is Hermitian, we sample angles only up to *π* and not 2*π*. The coefficients will be the same regardless. In the three-dimensional case, we choose to keep the angular sampling the same, and thus we cannot exploit the Hermitianity again in the z-direction.
5. For the power spectrum, we precompute the radial binning function and save it in memory as a sparse matrix. For a grid of side *H*, dimension *d*, and NR radial bins, we have a matrix $P\in {{ \mathcal R }}^{({H}^{d},\mathrm{NR})}$ where *P*_*ij*_ is 1 if the *i*th pixel of the flattened the *d*-dimensional array falls into the *j*th bin. We also save the normalization needed, which does not depend on the input field.

The runtime of our statistic code is described in Table 4.

**Table 4:** Runtime of Our Statistics Code on an NVIDIA A100-SXM4-40 GB for Double Precision Arithmetic

	Time (ms)							
	2D				3D			
	*H* = 128		*H* = 256		*H* = 128		*H* = 256	
	*N* = 10	*N* = 100	*N* = 10	*N* = 100	*N* = 1	*N* = 5	*N* = 1	*N* = 5
Pk	0.5 ± 0.2	0.6 ± 0.2	0.6 ± 0.2	1.0 ± 0.5	2.7 ± 0.2	3.6 ± 0.2	18 ± 1	25 ± 1
Bk (4096 configs)	108 ± 6	285 ± 2	143 ± 5	1164 ± 8	390 ± 20	1860 ± 30	2900 ± 50	14100 ± 200
WST(NR = 4, NT = 4)	13 ± 2	14 ± 2	13 ± 2	30 ± 2	⋯	⋯	⋯	⋯
WST(NR = 8, NT = 8)	145 ± 6	146 ± 5	145 ± 4	168 ± 4	⋯	⋯	⋯	⋯
WST(NR = 4, NT = 4, NZ = 3)	⋯	⋯	⋯	⋯	103 ± 7	176 ± 4	284 ± 5	1188.8 ± 0.1
WST(NR = 8, NT = 8, NZ = 6)	⋯	⋯	⋯	⋯	5390 ± 60	5430 ± 20	5410 ± 30	12220 ± 20

## Appendix G Cross Check of Our Fisher Matrices

To confirm that we did not make a mistake in our analysis, we check our standard power spectrum Fisher matrix with the Fisher results published in Villaescusa-Navarro et al. (2020). We note that although very close, they are not numerically identical, and we suspect that this results from the binning choice of the power spectrum.

## Appendix H Check with an External Bispectrum

We do notice that small choices when implementing the FFT estimator (Sefusatti 2005; Watkinson et al. 2017) could alter the Fisher analysis results and could thus be important. Thus, it could be the case that only the bispectrum coefficients we obtained with *our* code show non-Gaussianity. If so, the *χ* ^2^ distributional test would only be detecting non-Gaussianity from our own products. To verify that the finding generalizes to previously published bispectra, in this case from the Quijote suite products (Villaescusa-Navarro et al. 2020), we apply the *χ* ^2^ distributional test here. As we see in Figure 15, we clearly detect the non-Gaussianity in this bispectrum.
